# Evolutionary game analysis of FinTech transformation: A social co-governance pattern of peer-to-peer lending market in China

**DOI:** 10.3389/fpsyg.2022.954132

**Published:** 2022-12-07

**Authors:** Qi Wang, Xin Liu, Chenghu Zhang

**Affiliations:** School of Economics and Finance, Xi'an Jiaotong University, Xi'an, China

**Keywords:** Financial Technology (FinTech), social co-governance, tripartite evolutionary game, transformation, peer-to-peer lending

## Abstract

Benign exit has become the main theme of the transformation in China's peer-to-peer (P2P) lending industry. To protect the interests of investors in the benign exit process, this paper proposes a social co-governance pattern using a tripartite evolutionary game model to capture the behavior strategies of P2P lending platforms, investors, and financial regulators. The results demonstrate that there are four evolutionary stable strategies for the game model, among which the positive disposal of P2P lending platforms, the participation of the investors, and the co-governance policy of financial regulators is the optimal strategy in the benign exit process. The results also show that the initial proportion of P2P lending platforms, investors, and financial regulators would significantly affect the convergence speed of the evolutionary stable strategy. The proposed social co-governance pattern would effectively safeguard the interests of investors if incentive, penalty, and reputation mechanisms are well-designed. This paper provides in-depth implications for protecting investors' interests in the transformation of the P2P lending industry and enhancing the sustainable development of the FinTech industry.

## Introduction

**Figure 1:**
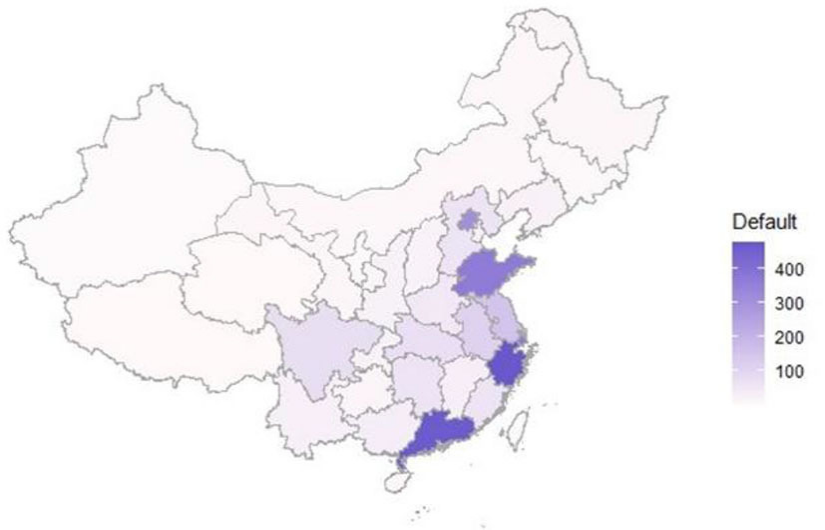
Geographical distribution of defaulted P2P lending platforms in China.

Digital technology, such as big data, block chain, and cloud computing, is quickly evolving in the third technological revolution (Li G. et al., 2022). The game-changing technological innovations have been triggered and introduced into Financial Technology (FinTech), transforming the way the financial industry operates and fulfilling customers' needs (Agarwal and Zhang, 2020; Brandl and Hornuf, 2020; Chen and Sivakumar, 2021; Weng and Luo, 2021; Lei et al., 2022). Taking its place as one of the most significant segments in the broad area of FinTech (Luther, 2019), peer-to-peer (P2P) lending has emerged as an infomediary platform that links up investors and borrowers to form debt–credit relationships *via* the Internet (Lee and Lee, 2012; Lin et al., 2013; An et al., 2022). Compared with the traditional financing channel, P2P lending platforms not only facilitate a convenient approach for small- and medium-sized enterprises to attain short-term loans (Feng et al., 2015) but also provide feasible investment options to investors for their idle capital (Bachmann et al., 2011; Wei and Lin, 2017). After the vigorous development of the last decade, the number of P2P lending platforms in China has reached 6,607 by 2022, with the total amount of loans exceeding 1.27 trillion dollars. Despite its merits, P2P lending has received strong criticism for its uncertain legal (Yang et al., 2018), regulatory arbitrage (Deng, 2022), and credit risks (Giudici et al., 2019). Numerous defaulted platforms occurred from time to time in the P2P lending market and may culminate in substantial financial losses, including fund-raising fraud, suspended operations, lost investment, and even business close-down (Fu et al., 2020). There is a remarkable increase in the number of defaulted P2P lending platforms in China (Yoon et al., 2019), and a growing default rate that achieves the highest point in history, i.e., 44.37% by 2021. The defaulted platforms are mainly located in the southeast and central regions of China, with the southeast region being the most severe (refer to Figure 1), which seriously infringes the interests of consumers and undermines the sustainability of the FinTech industry.

The increasing risks in the P2P lending industry have heightened the pressing need for financial regulators to develop policies to guide P2P lending platforms for transformation. In 2019, the Regulation and Rectification Office (RRO)^1^ issued the regulatory policy “Guidance on P2P lending platform disposition by classification and risk prevention,” urging P2P lending platforms to follow the guideline of benign exit.^2^ However, from a realistic point of view, the benign exit process of P2P lending platforms is not satisfactory concerning the protection of investors' interests. Hundreds of large-scale P2P lending platforms such as “*Tuandaiwang*,” “*Wanglibao*,” and “*Aiqianjin*,” which have been investigated by The Ministry of Public Security of China, resulted in billions of dollars in property losses for investors. Hence, there is an urgent need to address these problems by building up a benign exit pattern in the P2P lending industry to protect the legitimate rights and interests of investors, with respect to providing in-depth insights for the future sustainable development of the FinTech industry.

Since the P2P lending industry has shown irrational prosperity in the last decade due to the long-term inclusive policies in China (He and Li, 2021), thus the benign exit of P2P lending platforms is inseparable from the rigorous policy of financial regulators. However, the primary issue of the P2P lending platforms facing during the process of benign exit is the huge credit assignment with investors, which would be difficult to solve either by financial regulators or by investors. Thus, the co-governance of financial regulators, investors, and P2P lending platforms in the benign exist process can meet the interests of all stakeholders, which are the embodiment of corporate social responsibility (Jo and Harjoto, 2012; Kim et al., 2018; Wang et al., 2020). Compared with traditional regulation, the co-governance argues that the government, the market, and related stakeholders should be in equal positions to form a coordinated and effective network, in order to effectively distribute social benefits and ensure the maximization of social interests (Ackerman, 2004; Wu et al., 2018). Co-governance is more proactive and creative in the benign exit process of P2P lending platforms, which can drive investors to prevent P2P lending platforms from vicious exit through social supervision and encourage P2P lending platforms to comply with the law through industry self-regulation and market incentives. In addition, co-governance can improve the flexibility of regulation, broaden the applicability of policies, and reduce the cost of the benign exit process.

From the co-governance perspective, this study provides novel insights into the benign exit of P2P lending platforms and has three main contributions to filling gaps in the existing literature. First, the benign exit pattern of P2P lending platforms will be discussed from the perspective of co-governance, which can provide deeper implications for the research of defaulted P2P lending platforms. Second, we construct a tripartite evolutionary game to capture the behavioral strategies of P2P lending platforms, investors, and financial regulators, to develop a more effective co-governance pattern of the benign exist process. Third, the evolutionary stable strategy for enabling the benign exit of the P2P lending platform is determined. A sensitivity analysis of the influencing factors is performed to understand the effective conditions that can drive the tripartite evolutionary game model to its ideal status faster. This study provides policy implications by shedding light on the premise of interaction among P2P lending platforms, investors, and financial regulators and illustrates how to weigh strategies of the stakeholders to maximize payoffs and achieve a stable benign exit of P2P lending platforms.

The remainder of this paper is organized as below. Section Literature review reviews the literature. Section Problem description and basic assumptions describes the problem and establishes a tripartite evolutionary game model including P2P lending platforms, investors, and financial regulators. Section Stability analysis conducts numerical simulations to verify the theoretical results and sensitivity analysis to provide deeper insights into the studied problem. Section Numerical simulation presents a discussion of the results. Section Discussion summarizes the conclusions and puts forward relevant policy implications. Section Conclusion draws the conclusion.

## Literature review

This research is closely related to three streams of literature: the risk of P2P lending platforms and their regulation, the co-governance and its applications, and the application of evolutionary games in the research of P2P lending platforms.

### Defaulted platform and its regulation

Peer-to-peer lending platforms connect investors and borrowers *via* qualified third-party internet platforms, which not only facilitate a convenient approach for small and medium enterprises to get short-term loans but also provide feasible investment options to investors for their idle capital (Bachmann et al., 2011). However, with the main technological drivers, i.e., big data analytics, artificial intelligence, and block chain technology, risks arise with the development of the most important financial technologies, i.e., peer-to-peer lending (Giudici, 2018; Aldasoro et al., 2022). Particularly, the P2P lending platforms in China are prone to default due to the absence of stringent market regulations (Zhang and Wang, 2019) and low entry barriers (Chen and Tsai, 2017), causing serious damage to the interests of consumers and the stability of the industry, which has gradually aroused scholars' concerns (Emekter et al., 2015; Li et al., 2016; Yang and Luo, 2017; Liu et al., 2018; Xia et al., 2019). Prior studies mainly focused on the identification of the key factors that affect platform risk and the application of models to predict risk to mitigate the platform risks in P2P lending industry (Giudici et al., 2020). The impacts of platform characteristics and macro-financial environment factors on the default risk of P2P lending platforms are examined (Yoon et al., 2019). It is demonstrated that P2P lending platforms that closed down as a result of liquidity issues were usually due to a lack of high-quality risk management techniques (Liu et al., 2019). From the investors' side, it is found that the higher the level of risk aversion of the investors, the higher the level of risks of the P2P lending platform (Yan et al., 2018; Cheng and Guo, 2020). By digging deep into the very nature of the platforms, it is also shown that the impact of network effects in peer-to-peer lending platforms would increase the opportunities and risks for both investors and borrowers (Chen et al., 2022).

A large and growing body of literature has investigated the regulation of defaulted P2P lending platforms. It is argued that P2P lending is an example of how modern technology enables the integration of a range of economic functions, therefore, a new approach to market regulation is warranted which is more consistent with emerging institutional arrangements (Davis, 2016). It is pointed out that the regulation of P2P lending should be consistent with leveraged information mechanisms to reduce information asymmetry and market friction and ensure market transparency, competition, and fair pricing (Yang et al., 2018). It is also pointed out that the financial regulators should first motivate and then regulate the P2P lending platforms (Zhang and Wang, 2019). In comparison to the regulatory regime of P2P lending markets in the US, the UK, and Japan, the extent to which the new regulatory regime was likely to reduce the default of P2P lending platforms to protect the interests of the investors is examined (Huang, 2018). It is concluded that a “national bank charter” was the best way to ensure greater regulation of the P2P lending industry, provide sufficient incentives for investors, reduce systemic risk, and allow for greater regulatory knowledge of related institutions to ensure their compliance of consumer protection laws (Luther, 2019).

### Co-governance and its applications

In the late twentieth century, the high welfare policies of developed countries resulted in overstaffed and inefficient government agencies (Offe, 1984). It is noted that the government, the market, and the social actors are supposed to be in equal positions and form a coordinated and effective network in order to more effectively distribute and ensure the maximization of social benefits (Gelatt, 1992). On this basis, an inclusive and flexible concept of co-governance was formed. The co-governance was defined as an approach in which a mixture of instruments is brought to bear on a specific problem, emphasizing the coordination between public and private agents in the regulatory process (Eijlander, 2005). According to a prior study (Bartle and Vass, 2005), co-governance may arise in the process of creating regulatory rules by incorporating the opinions of the government, non-governmental organizations, market players, individuals, and other stakeholders. Co-governance in enforcement involves all modes of regulation in which regulations are designed and set by public authorities and enforced by the coordinated actions of public authorities and regulated firms (Rouvière and Ca Swell, 2012). It is concluded that the degree of cooperation and competition depends on the existing regulatory arrangements, the congruence of goals of the different actor groups, and the institutionalization of industrial relations (Tosun et al., 2016).

Researchers have explored these potential complementarities and gains from coordination in many different areas of regulation. In the environmental protection landscape, the legal and institutional frameworks of environmental co-governance were constructed to adopt co-construction, co-governance, and the sharing of innovative social governance patterns (Birnbaum, 2016; Iaione, 2016; Ko et al., 2019; Abdullah et al., 2020; Jin et al., 2020; Xu et al., 2020). In more recent years, the concept of co-governance has gradually aroused policy makers' attention in the field of food safety regulation, arguing that co-governance is a kind of societal-wide innovation that integrates diverse resources and efforts from multiple stakeholders including government, industry, and social forces for better and sustainable development of an economy's food institution and system (Martinez et al., 2007; Wu et al., 2018; Chen and Li, 2019; Chen and Wu, 2019; Meng et al., 2021; Pan et al., 2021; Yan et al., 2021). It is found that a significant degree of cooperation between private regulators and public supervisors was the key to ensuring the effectiveness of regulation and pointed out that public supervision and enforcement must be responsive to the peculiarities of co-governance arrangements (Cherednychenko, 2016). Few works of literature pay attention to the application of co-governance in the E-commerce field. Through an evolutionary game model among the government, E-commerce platforms, and rights holders, it is found that reasonable adjustment of the reward and punishment measures of government supervisory agencies can produce positive guidance to platform and operators, and the related social environment, social benefits, the sense of acquisition by the government, platforms and rights holders can be strengthened (Li J. et al., 2022).

### Application of evolutionary games in the P2P lending market

Compared to classic game theory, evolutionary game theory has the merits of considering the bounded rationality of the players with the ability to keep learning, adapt to the market environment, and adjust their strategies, which can describe the dynamic process of decision-making theoretically (Taylor, 1979; Smith, 1988). Particularly, evolutionary game theory was widely used to allow a deeper insight into the bounded rationality of P2P lending platforms, investors, regulators, and other stakeholders. Previous research has established evolutionary game models among regulatory authorities, P2P lending platforms, and borrowers to evaluate the strict supervision strategy of the P2P lending platform (You et al., 2021) and determine certain conditions when the three players will converge on the strategies of positive supervision, self-discipline operation, and compliance, respectively (Chunsheng, 2020). An evolutionary game model was established between local governments and P2P lending platforms regarding the benign exit of P2P lending platforms in China, and it is noted that the two players can converge to the optimal equilibrium of “incentives and benign exits” under certain conditions (Zhang et al., 2020). An evolutionary game model of different regulatory stages was established to analyze the optimal state of the P2P lending market structure under the strengthening supervision and shrinking industry (Peng et al., 2020). Evolutionary game models were also developed in several lines of research to help understand the risk supervision of P2P lending platforms (Gu et al., 2018), to investigate the influence pathway of the guarantee mechanism on users' participation (Weng and Luo, 2021), and to analyze the risk preference behavior of lenders and the credit choice of borrowers (Liu and Xia, 2017).

The literature presented, thus, far provides sufficient studies on the default risk regulation of P2P lending platforms. Undoubtedly, these studies have laid a solid foundation for understanding the default risk of P2P lending platforms and provide practical implications to policy makers. However, few scholars have been able to draw on any rigorous research into the benign exit process of P2P lending platforms, and the crucial role of investors in the regulation of P2P lending platforms has been ignored. The study would have been more useful if a co-governance pattern in the benign exit process of P2P lending platforms in China was considered. To address this problem, this paper established a tripartite evolutionary game model including P2P lending platforms, investors, and financial regulators, studied the formulation of the co-governance pattern, concentrated on the analysis of the influencing factors of the co-governance pattern on the evolution stability strategy (ESS; Smith and Price, 1973).

## Problem description and basic assumptions

### Problem description

Based on a co-governance framework, the P2P lending platforms, investors, and financial regulators are the main players in the benign exit process of P2P lending platforms. P2P lending platforms need to comply with the benign exit rules, address their debt risks by returning credit assignments, and meet the demands of investors and financial regulators. Moreover, the P2P lending platforms are supposed to protect the legitimate rights and interests of investors to the maximum extent in a timely manner.

The investors, however, are not only the direct stakeholders of the benign exit of P2P lending platforms but also the direct beneficiaries of the regulation of P2P lending platforms. Hence, it is essential for investors to participate in the co-governance of the benign exit process. Specifically, the investors' supervision of P2P lending platforms and the performance assessment of financial regulators would play a significant role in their strategies.

**Figure 2:**
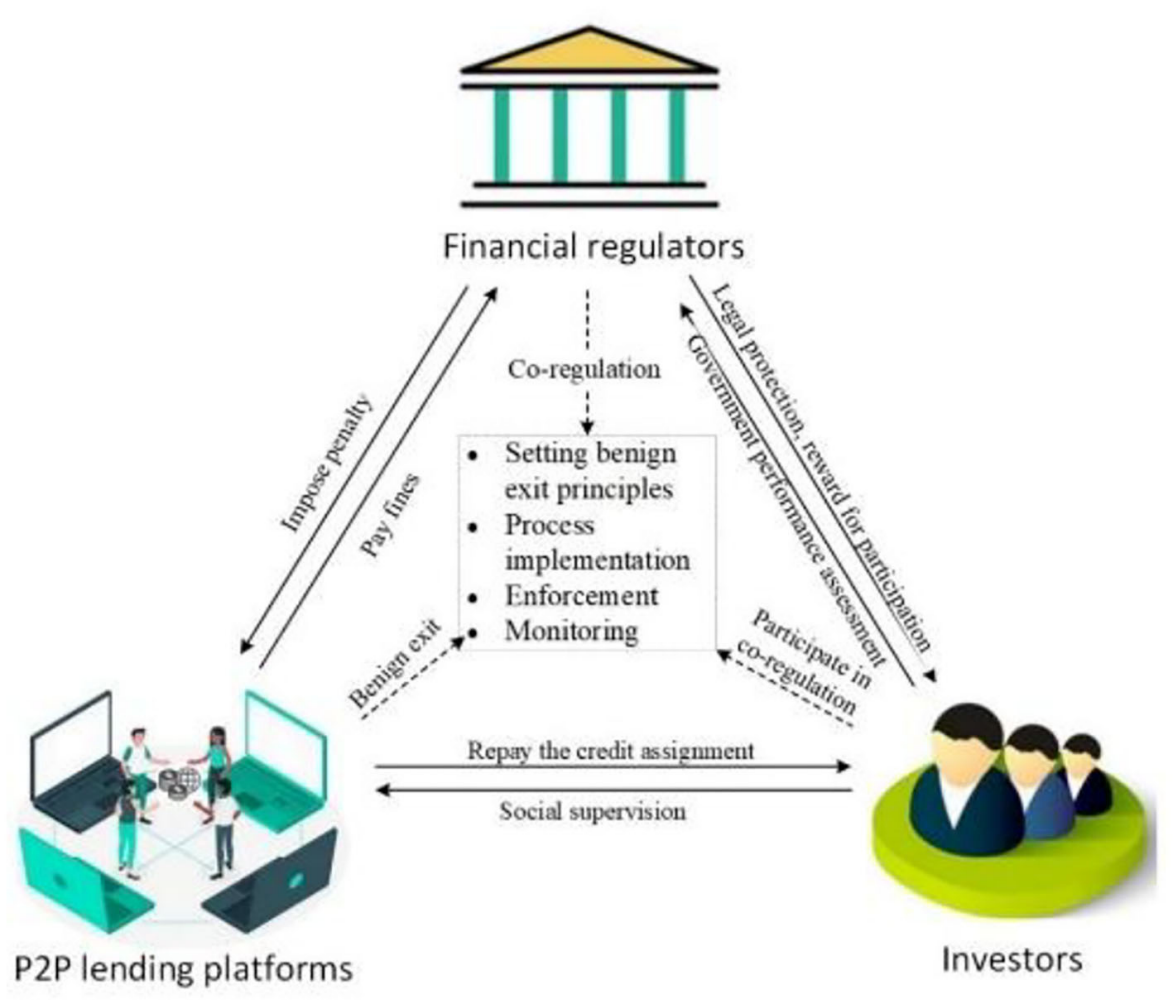
Co-governance scheme of P2P lending platforms' benign exit.

There is strong evidence that financial regulators play a crucial role in regulating P2P lending platforms. The financial regulators, which aim to accelerate the benign exit process and protect the rights and interests of investors, undertake multiple tasks such as imposing a penalty on P2P lending platforms that do not comply with the benign exit rules and rewarding investors' positive engagement in co-governance. Effective financial regulation will have a conductive role in restraining the operations strategy of P2P lending platforms and protecting the interests of investors. Otherwise, it will jeopardize the public assessment of financial regulators' performance. Above all, the co-governance pattern of benign exit of P2P lending platforms is established based on the interaction of P2P lending platforms, investors, and financial regulators, as shown in Figure 2.

In the co-governance pattern, P2P lending platforms, investors, and financial regulators are supposed to coordinate and cooperate in building principles, ensuring process implementation, enforcement, and supervision to accelerate the benign exit process. Therefore, the repayment ratio of the non-performing assets will be increased, and the asset degradation will be reduced. Specifically, building principles refer to verifying the asset and capital of P2P lending platforms and forming benign exit principles. Process implementation refers to putting these principles into real practice. Enforcement refers to ensuring the benign exit of P2P lending platforms in compliance with laws and regulatory policies. Supervision refers to the ongoing investigation of the whole process of benign exit of P2P lending platforms.

### Payoffs of three participants

#### Scenario with co-governance regulation

This sub-section will discuss the expected payoffs of P2P lending platforms, investors, and financial regulators based on the co-governance pattern.

##### P2P lending platforms' expected payoffs

When P2P lending platforms adopt the “positive disposal” strategy and investors adopt the “participating in co-governance” strategy, then the P2P lending platforms will receive the reputation benefits from the investors. Thus, their expected payoffs are equal to −*C*_*P*1_ + ψ_*P*1_ − θ_1_*pD*(1 + *i*). If investors adopt the “not participating in co-governance” strategy, then the P2P lending platforms cannot gain the reputation benefits from investors, and the expected payoffs are equal to −*C*_*P*1_ − θ_1_*pD*(1 + *i*). When P2P lending platforms adopt the “negative disposal” strategy, their costs will be reduced from *C*_*P*1_ to *C*_*P*2_; however, once this behavior is found by the financial regulators, they will suffer a penalty, in this case, their expected payoffs in case investors adopt “participating in co-governance” or “not participating in co-governance” are equal to −*C*_*P*2_ − α*F* − αθ_2_*pD*(1 + *i*) − ψ_*P*2_ and −*C*_*P*2_ − β*F* − βθ_2_*pD*(1 + *i*), respectively.

##### Investors' expected payoffs

Investors can obtain a fraction of credit assignment from the P2P lending platforms plus the rewards from the financial regulators when they adopt the “participating in co-governance” strategy, thus, their expected payoffs in case P2P lending platforms adopt “positive disposal” and “negative disposal” strategy are equal to −(1−θ_1_)*pD*(1+*i*)+*R*_*L*_−*C*_*L*_/*D* and−α(1−θ_2_)*pD*(1 + *i*) + *R*_*L*_ − *C*_*L*_/*D*, respectively. If the investors adopt the “not participating in co-governance” strategy, they will not gain the rewards from the financial regulators; in this case, their payoffs will be dependent on the P2P lending platforms' strategy choice and the regulatory efficiency of the financial regulators, thus, the expected payoffs of investors in case P2P lending platforms adopt “positive disposal” and “negative disposal” strategy will be equal to −(1−θ_1_)*pD*(1+*i*) and−β(1−θ_2_)*pD*(1+*i*), respectively.

##### Financial regulators' expected payoff

When the financial regulators adopt the “co-governance regulation” strategy, they will incur an initiative cost to set up the social co-governance pattern. In this case, when P2P lending platforms adopt a “positive disposal” strategy and investors adopt a “co-governance” strategy, then the expected payoffs of the financial regulators are equal to ψ_*R*1_ + ψ_*W*1_ − *M*_*R*_ − *C*_*R*1_ − *R*_*L*_. If investors adopt the “not participating in co-governance” strategy, then the expected payoffs of the financial regulators are equal to ψ_*W*1_ − *M*_*R*_ − *C*_*R*1_. When P2P lending platforms adopt a “negative disposal” strategy, there may be a social welfare loss in accordance with the regulation efficiency. In this case, if investors adopt a “participating in co-governance” strategy, then the financial regulators will be required to pay rewards to the investors; in this case, the expected payoffs of financial regulators are equal to ψ_*R*1_+α*F*−*M*_*R*_−*C*_*R*1_−*R*_*L*_−(1−α)ψ_*W*2_. If investors adopt the “not participating in co-governance” strategy, the payoffs of financial regulators will be equal to β*F* − *M*_*R*_ − *C*_*R*1_ − (1 − β)ψ_*W*2_. Table 1 shows the payoff matrix of three participants with co-governance regulation.

**Table 1:** The payoff matrix of the tripartite game.

**P2P lending platforms**	**Investors**	**Financial regulators**	
		**Co-governance (z)**	**Traditional regulation (1−*z*)**
Positive exit (*x*)	Participating in co-governance (*y*)	(α_1_, β_1_, γ_1_)	(α_2_, β_2_, γ_2_)
	Not participating in co- regulation (1−*y*)	(α_3_, β_3_, γ_3_)	(α_4_, β_4_, γ_4_)
Negative exit (1−*x*)	Participating in co- regulation (*y*)	(α_5_, β_5_, γ_5_)	(α_6_, β_6_, γ_6_)
	Not participating in co- regulation (1−*y*)	(α_7_, β_7_, γ_7_)	(α_8_, β_8_, γ_8_)

#### Scenario with traditional regulation

This sub-section will discuss the expected payoffs of P2P lending platforms, investors, and financial regulators in the scenario with traditional regulation.

##### P2P lending platforms' expected payoffs

When P2P lending platforms adopt the “positive disposal” strategy, their expected payoffs are the same as in the scenario with co-governance regulation. It should be noted that there is no direct causal relationship between the regulation type and P2P lending platforms' reputation benefits, their reputation benefits are associated with the investors' strategy choice. When P2P lending platforms adopt a “negative regulation” strategy, they will suffer a penalty from the financial regulators in accordance with the regulation efficiency, thus, their expected payoffs in case investors adopt “participating in co-governance” or “not participating in co-governance” are equal to −*C*_*P*2_−α*F*−αθ_2_*pD*(1+*i*)−ψ_*P*2_ and −*C*_*P*2_−β*F*−βθ_2_*pD*(1+*i*), respectively.

##### Investors' expected payoffs

Compared with the scenario with co-governance, whether the investors adopt the “participating in co-governance” strategy or “not participating in co-governance” strategy, they will not gain a reward from the financial regulators, thus, when the investors adopt the “participating in co-governance” strategy, the expected payoffs of investors in case the P2P lending platforms adopt “positive disposal” or “negative disposal” strategy are equal to −(1−θ_1_)*pD*(1+*i*)−*C*_*L*_/*D* and −*pD*(1+*i*)−*C*_*L*_/*D*, respectively. When investors adopt the “not participating in co-governance” strategy, the expected payoffs of investors in case the P2P lending platforms adopt a “positive disposal” or “negative disposal” strategy are equal to −(1−θ_1_)*pD*(1+*i*) and −*pD*(1+*i*), respectively.

##### Financial regulators' expected payoffs

When P2P lending platforms adopt the “positive disposal” strategy, the financial regulators will gain a social welfare improvement, thus, the expected payoffs of the financial regulators, in case investors adopt the “participating in co-governance” or “not participating in co-governance” strategy, are equal to −_*C*_*R*2_ + ψ*W*1_ − ψ_*R*2_ and ψ_*W*1_ − *C*_*R*2_, respectively. Moreover, when P2P lending platforms adopt the “negative disposal” strategy, the financial regulators will not find the P2P lending platform's “negative disposal” behavior in this scenario, thus, they will suffer a social welfare loss, then the expected payoffs of the financial regulators in case investors adopt “participating in co-governance” or “not participating in co-governance” strategy, are equal to −*C*_*R*2_ − ψ_*W*2_ − ψ_*R*2_ and −*C*_*R*2_ − ψ_*W*2_, respectively. Table 2 presents the specific payoff matrix of the three participants.

**Table 2:** The specific payoff of P2P lending platforms, investors, and financial regulators.

**Payoff**	**P2P lending platforms**	**Investors**	**Financial regulators**
(α_1_, β_1_, γ_1_)	−*C*_*P*1_ + ψ_*P*1_ − θ_1_*pD*(1 + *i*)	−(1 − θ_1_)*pD*(1 + *i*) + *R*_*L*_ − *C*_*L*_	ψ_*R*1_ + ψ_*W*1_ − *M*_*R*_ − *C*_*R*1_ − *R*_*L*_
(α_2_, β_2_, γ_2_)	−*C*_*P*1_ + ψ_*P*1_ − θ_1_*pD*(1 + *i*)	−(1 − θ_1_)*pD*(1 + *i*) − *C*_*L*_	−*C*_*R*2_ + ψ_*W*1_ − ψ_*R*2_
(α_3_, β_3_, γ_3_)	−*C*_*P*1_ − θ_1_*pD*(1 + *i*)	−(1 − θ_1_)*pD*(1 + *i*)	ψ_*W*1_ − *M*_*R*_ − *C*_*R*1_,
(α_4_, β_4_, γ_4_)	−*C*_*P*1_ − θ_1_*pD*(1 + *i*)	−(1 − θ_1_)*pD*(1 + *i*)	ψ_*W*1_ − *C*_*R*2_
(α_5_, β_5_, γ_5_)	−*C*_*P*2_ − α*F* − αθ_2_*pD*(1 + *i*) − ψ_*P*2_	−α(1 − θ_2_)*pD*(1 + *i*) + *R*_*L*_ − *C*_*L*_	ψ_*R*1_ + α*F* − *M*_*R*_ − *C*_*R*1_ − *R*_*L*_ − (1 − α)ψ_*W*2_
(α_6_, β_6_, γ_6_)	−*C*_*P*2_ − ψ_*P*2_	−*pD*(1 + *i*) − *C*_*L*_	−*C*_*R*2_ − ψ_*W*2_ − ψ_*R*2_
(α_7_, β_7_, γ_7_)	−*C*_*P*2_ − β*F* − βθ_2_*pD*(1 + *i*)	−β(1 − θ_2_)*pD*(1 + *i*)	β*F* − *M*_*R*_ − *C*_*R*1_ − (1 − β)ψ_*W*2_
(α_8_, β_8_, γ_8_)	−*C*_*P*2_	−*pD*(1 + *i*)	−*C*_*R*2_ − ψ_*W*2_

## Stability analysis

### Basic assumptions and model parameters

To analyze the defined problem, the main assumptions are summarized as follows:

**Assumption 1:** The participants in the game process include P2P lending platforms, investors, and financial regulators, as well as all of whom are bounded rational.

**Assumption 2:** The strategy set of P2P lending platforms is {positive disposal, negative disposal}, the strategy set of Investors is {participating in co-governance, not participating in co-governance}, and the strategy set of financial regulators is {co-governance regulation, traditional regulation}.

**Assumption 3:** The proportion of individuals in P2P lending platforms that adopt “positive disposal” strategy is *x*, and the proportion of individuals in P2P lending platforms that adopt “negative disposal” strategy is 1 − *x*. The proportion of individuals in investors that adopt “participating in co-governance” strategy is *y*, and the proportion of individuals in investors that adopts “not participating in co-governance” strategy is 1 − *y*. The proportion of individuals in financial regulators that adopt “co-governance regulation” strategy is *z*, and the proportion of individuals in financial regulators that adopt “traditional regulation” strategy is 1 − *z*.

**Assumption 4:** During the process of the game, every player in the game wants to get their maximum benefits. However, they cannot get their optimal strategies in a game due to their bounded rationality. They can change their strategies through learning and observing in the continuous games until achieving their stable states.

**Assumption 5:** When P2P lending platforms adopt the “negative disposal” strategy, they will pay less costs. However, once the “negative disposal” behavior is investigated by the financial regulators, the financial regulators will impose penalty on P2P lending platforms to push them to adopt the “positive disposal” strategy.

The symbols and connotations of the parameters used in the model are listed in Table 3.

**Table 3:** Symbols and notations of the parameters in the model.

**Parameters**	**Connotations**
*C*_*P*1_	The cost of P2P lending platforms when adopting “positive disposal” strategy.
*C*_*P*2_	The cost of P2P lending platforms when adopting “negative disposal” strategy.
θ_1_	The proportion of credit assignment to the investors when P2P lending platforms adopting “positive disposal” strategy.
θ_2_	The proportion of creditor's rights transferred to the investors when P2P lending platforms adopting “negative disposal” strategy.
*F*	The penalty of financial regulators to P2P lending platforms when P2P lending platforms adopt “negative disposal” strategy.
ψ_*P*1_	The reputation benefits of P2P lending platforms.
ψ_*P*2_	The reputation loss of P2P lending platforms.
*R*_*L*_	The rewards of financial regulators to investors when investors adopt “participating in co-governance” strategy.
*C*_*L*_	The cost of investors when adopting “participating in co-governance” strategy.
*D*	The centrality degree of investors.
*p*	The investment volume of investors.
*i*	The interest rates.
*C*_*R*1_	The cost of financial regulators when adopting “co-governance regulation.”
*C*_*R*2_	The cost of financial regulators when adopting “traditional regulation” strategy, *C*_*R*2_ < *C*_*R*1_.
α	The regulatory efficiency of co-governance regulation.
β	The regulatory efficiency of traditional regulation.
ψ_*w*1_	The social welfare improvement when P2P lending platforms adopt “positive disposal” strategy.
ψ_*w*2_	The social welfare loss when P2P lending platforms adopt “positive disposal” strategy.
ψ_*R*1_	The reputation benefits of financial regulators when adopting “co-governance regulation” strategy.
ψ_*R*2_	The reputation loss of financial regulators when adopting “traditional regulation” strategy.
*M*_*R*_	The initiative cost of financial regulators to set up the co-governance regulation pattern.

### Replicated dynamic equation

Set the expected payoff of P2P lending institutions' “positive disposal” strategy as *U*_*P*1_, the expected payoff of P2P lending institutions' “negative disposal” strategy as *U*_*P*2_, the average expected payoff of P2P lending institutions as ${\bar{U}}_{P}$. The expected payoff of investors' “participating in co-governance” strategy as *U*_*L*1_, the expected payoff of investors' “not participating in co-governance” strategy as *U*_*L*2_, the average expected payoff of investors as ${\bar{U}}_{L}$. The expected payoff of financial regulators' “co-governance regulation” strategy as *U*_*R*1_, the expected payoff of financial regulators' “traditional regulation” strategy as *U*_*R*2_, the average expected payoff of financial regulators as ${\bar{U}}_{R}$.

According to Table 2, the expected payoffs that P2P lending platforms gain when they adopt the “positive disposal” strategy is as follows:


(1) $$\begin{array}{l} U_{P1}=yz[-C_{P1}+\psi_{P1}-\theta_{1}pD(1+i)]\\ \;+y(1-z)[-C_{P1}+\psi_{P1}-\theta_{1}pD(1+i)]\;+(1-y)z[-C_{P1}\\ \;-\theta_{1}pD(1+i)]+(1-y)(1-z)[-C_{P1}-\theta_{1}pD(1+i)] \end{array}$$

The expected payoffs that P2P lending institutions gain when they choose the “negative disposal” strategy is as follows:


(2) $$\begin{array}{l} U_{P2}=yz[-C_{P2}-\alpha F-\alpha \theta_{2}pD(1+i)-\psi_{P2}]\\ \;+y(1-z)(-C_{P2}-\psi_{P2})\;+(1y)z[-C_{P2}-\beta F\\ \;-\beta \theta_{2}pD(1+i)]+(1-y)(1-z)(-C_{P2}) \end{array}$$

Accordingly, the average expected payoff of P2P lending platforms is as follows:


(3) $${\bar{U}}_{P}=x\Pi_{P1}+(1-x)\Pi_{P2}$$

Taking the proportion of the “positive disposal” strategy as an example, the replicated dynamic equation of P2P lending platforms can be expressed as follows:


(4) $$\begin{array}{l} F(x)=\frac{dx}{dt=x(}U_{P1}-{\bar{U}}_{P})\\ \;=x(1-x)\{(\psi_{P1}+\psi_{P2})y+[\beta F+\beta \theta_{2}pD(1+i)]z\\ \;+(\alpha -\beta )[F+\theta_{2}pD(1+i)]yz+C_{P2}-C_{P1}\\ \;-\theta_{1}pD(1+i)\} \end{array}$$

The expected payoffs that investors gain when they adopt the “participating in co-governance” strategy is as follows:


(5) $$\begin{array}{l} U_{L1}=xz[-(1-\theta_{1})pD(1+i)+R_{L}-C_{L}/D]\\ \;+x(1-z)[-(1-\theta_{1})pD(1+i)-C_{L}/D]\\ \;+(1-x)z[-\alpha (1-\theta_{2})pD(1+i)+R_{L}-C_{L}/D]\\ \;+\text{ (1}-x\text{)(1}-z\text{)[}-pD\text{(1 }+\;i\text{) }-C_{L}\text{/}D\text{]} \end{array}$$

The expected payoffs that investors gain when they adopt the “not participating in co-governance” strategy is as follows:


(6) $$\begin{array}{l} U_{L2}=xz[-(1-\theta_{1})pD(1+i)]+x(1-z)[-(1-\theta_{1})pD(1+i)]\\ \;+(1-x)z[-\beta (1-\theta_{2})pD(1+i)]\\ \;+\text{ (1}-x\text{)(1}-z\text{)[}-pD\text{(1}+i\text{)]} \end{array}$$

Similarly, the average expected payoff of investors is as follows:


(7) $${\bar{U}}_{L}=yU_{L1}+(1-y)U_{L2}$$

Accordingly, the replicated dynamic equation of investors adopting the “participating in co-governance” strategy can be expressed as follows:


(8) $$\begin{array}{l} F(y)=\frac{dy}{dt=y(}U_{L1}-{\bar{U}}_{L})\;=y(1-y)\{[R_{L}-(\alpha -\beta )(1\\ \;-\theta_{2})pD(1+i)]z+(\alpha -\beta )(1-\theta_{2})pD(1+i)xz\\ \;-C_{L}/D\} \end{array}$$

Additionally, the expected payoff that financial regulators gain when they adopt the “co-governance regulation” strategy is as follows:


(9) $$\begin{array}{l} U_{R1}=xy\left(\psi_{R1}+\psi_{W1}-M_{R}-C_{R1}-R_{L}\right)\\ \;+x\left(1-y\right)\left(\psi_{W1}-M_{R}-C_{R1}\right)\\ \;+(1-x)y[\psi_{R1}+\alpha F-M_{R}-C_{R1}-R_{L}-(1-\alpha )\psi_{W2}]\\ \;+(1-x)(1-y)[\beta F-M_{R}-C_{R1}-(1-\beta )\psi_{W2}] \end{array}$$

The expected payoff that financial regulators gain when they adopt the “traditional regulation” strategy is as follows:


(10) $$\begin{array}{l} U_{R2}=xy(-C_{R2}+\psi_{W1}-\psi_{R2})+x(1-y)(\psi_{W1}-C_{R2})\;\\ \;+(1-x)y(-C_{R2}-\psi_{W2}-\psi_{R2})\\ \;+(1-x)(1-y)(-C_{R2}-\psi_{W2}) \end{array}$$

The average expected payoff of the financial regulators is as follows:


(11) $${\bar{U}}_{R}=zU_{R1}+(1-z)U_{R2}$$

Similarly, the following replicated dynamic equation can be obtained when the financial regulators adopt a “co-governance regulation” strategy:


(12) $$\begin{array}{l} \frac{F(z)=dz}{dt}=z\left(U_{R1}-{\bar{U}}_{R}\right)\;=z(1-z)\{-\beta \left(F+\psi_{W2}\right)x\\ \;+\left[\psi_{R1}+\psi_{R2}+\left(\alpha -\beta \right)\left(F+\psi_{W2}\right)-R_{L}\right]y\\ \;-[(\alpha -\beta )(F+\psi_{W2})]xy+C_{R2}+\beta (F+\psi_{W2})\\ \;-C_{R1}-M_{R} \end{array}$$

Due to the limited rationality of P2P lending platforms, investors, and financial regulators, it is difficult for them to make the best choice in a game. Therefore, Equations 4, 8, 12 can be considered as indicating an evolutionary process, forming a tripartite replicated dynamic system. Maybe, with the development of iterations, P2P lending platforms, investors, and financial regulators can find strategies to maximize their benefits, and eventually, develop an evolutionary stable strategy (ESS).

### Evolutionary stable strategies

As mentioned in Section Basic assumptions and model parameters, the whole game is constantly evolving, hence, the proportions of any strategies chosen by P2P lending platforms, investors, and financial regulators are time-dependent and can be expressed as *x*(*t*), *y*(*t*), and *z*(*t*) ∈ [0, 1], respectively. Thus, the solution of the replicated dynamic system, consisting of Equations 4, 8, 12, is as follows: [0, 1] × [0, 1] × [0, 1].

Obviously, when all the dynamic equations equal 0, which means that the whole dynamic system will tend to be stable, the P2P lending platforms, investors, and financial regulators have been able to select the optimal strategy. Thus, the equilibrium points of the tripartite game model can be calculated in the following way:


(13-1) $$\begin{array}{l} F(x)=\frac{dx}{dt=x(}U_{P1}-{\bar{U}}_{P})=x(1-x)\{(\psi_{P1}+\psi_{P2})y\\ \;+[\beta F+\beta \theta_{2}pD(1+i)]z+(\alpha -\beta )[F\\ \;+\theta_{2}pD(1+i)]yz+C_{P2}-C_{P1}-\theta_{1}pD(1+i)\}\\ \;=0 \end{array}$$

(13-2) $$\begin{array}{l} F(y)=\frac{dy}{dt=y(}U_{L1}-{\bar{U}}_{L})\\ \;=y(1-y)\{\left[R_{L}-\left(\alpha -\beta \right)\left(1-\theta_{2}\right)pD\left(1+i\right)\right]z\\ \;+(\alpha -\beta )(1-\theta_{2})pD(1+i)xz-C_{L}/D\}\\ \;=0 \end{array}$$

(13-3) $$\begin{array}{l} \frac{F\left(z\right)=dz}{dt}=z\left(U_{R1}-{\bar{U}}_{R}\right)=z(1-z)\{-\beta (F+\psi_{W2})x\\ \;+[\psi_{R1}+\psi_{R2}+(\alpha -\beta )(F+\psi_{W2})-R_{L}]y\\ \;-[(\alpha -\beta )(F+\psi_{W2})]xy+C_{R2}+\beta (F+\psi_{W2})\\ \;-C_{R1}-M_{R}\}=0 \end{array}$$

The equilibrium points can be identified easily by solving Equations 13-1, 13-2, and 13-3. Among these, there are eight special equilibrium points: *E*_1_(0, 0, 0),*E*_2_(1, 0, 0),*E*_3_(0, 1, 0),*E*_4_(0, 0, 1),*E*_5_(1, 0, 1),*E*_6_(1, 1, 0), *E*_7_(0, 1, 1), and *E*_8_(1, 1, 1). All stakeholders adopt a pure strategy in each of these equilibrium points, which constitute the boundary of the domain. In addition, there may exist other mixed strategy equilibrium points by solving Equation 14.


(14) $$\left\{ \begin{array}{l} \left(\psi_{P1}+\psi_{P2}\right)y+\left[\beta F+\beta \theta_{2}pD\left(1+i\right)\right]z\\ +\left(\alpha -\beta \right)\left[F+\theta_{2}pD\left(1+i\right)\right]yz+C_{P2}-C_{P1}\\ -\theta_{1}pD\left(1+i\right)=0\\ \left[R_{L}-\left(\alpha -\beta \right)\left(1-\theta_{2}\right)pD\left(1+i\right)\right]z\\ +\left(\alpha -\beta \right)\left(1-\theta_{2}\right)pD\left(1+i\right)xz-\frac{C_{L}}{D}=0\\ -\beta (F+\psi_{W2})x+[\psi_{R1}+\psi_{R2}+(\alpha -\beta )(F+\psi_{W2})-R_{L}]y\\ -[(\alpha -\beta )(F+\psi_{W2})]xy+C_{R2}+\beta (F+\psi_{W2})-C_{R1}\\ -M_{R}=0 \end{array}\right.$$

However, according to Friedman's evolutionary game theory (Friedman, 1998), only if the equilibrium points simultaneously satisfy both a strict Nash equilibrium and a pure strategy Nash equilibrium, then they will turn into an asymptotically stable equilibrium, and thus the equilibrium point will be an ESS. Consequently, we only need to analyze the stability of the eight pure strategy Nash equilibrium points.

Moreover, according to Wainwright (1989) and Lyapunov (1992), the asymptotic stability at the equilibrium point can be evaluated by analyzing the eigenvalues of the Jacobian matrices of the system, whereby the necessary and sufficient condition for the asymptotic stability of the system is that all the eigenvalues of the Jacobian matrix are negative. Thus, the Jacobian matrix of the tripartite dynamic game can be calculated as follows:


(15) $$J=\left(\begin{array}{ccc} \frac{\partial F(x)}{\partial x} & \frac{\partial F(x)}{\partial y} & \frac{\partial F(x)}{\partial z}\\ \frac{\partial F(y)}{\partial x} & \frac{\partial F(y)}{\partial y} & \frac{\partial F(y)}{\partial z}\\ \frac{\partial F(z)}{\partial x} & \frac{\partial F(z)}{\partial y} & \frac{\partial F(z)}{\partial z} \end{array}\right)=\left(\begin{array}{ccc} J_{11} & J_{12} & J_{13}\\ J_{21} & J_{22} & J_{23}\\ J_{31} & J_{32} & J_{33} \end{array}\right)$$

where


(16) $$\begin{array}{l} J_{11}=(1-2x)\{(\psi_{P1}+\psi_{P2})y+[\beta F+\beta \theta_{2}pD(1+i)]z\\ \;+(\alpha -\beta )[F+\theta_{2}pD(1+i)]yz+C_{P2}-C_{P1}\\ \;-\theta_{1}pD(1+i)\} \end{array}$$

(17) $$\begin{array}{l} J_{12}=x(1-x)\{\psi_{P1}+\psi_{P2}\\ \;+(\alpha -\beta )[F+\theta_{2}pD(1+i)]z\} \end{array}$$

(18) $$\begin{array}{l} J_{13}=x(1-x)\{\beta F+\beta \theta_{2}pD(1+i)\\ \;+(\alpha -\beta )[F+\theta_{2}pD(1+i)]y\} \end{array}$$

(19) $$J_{21}=y(1-y)(\alpha -\beta )(1-\theta_{2})pD(1+i)z$$

(20) $$\begin{array}{l} J_{22}=(1-2y)\{[R_{L}-(\alpha -\beta )(1-\theta_{2})pD(1+i)]z\\ \;+(\alpha -\beta )(1-\theta_{2})pD(1+i)xz-C_{L}/D\} \end{array}$$

(21) $$\begin{array}{l} J_{23}=y(1-y)[R_{L}-(\alpha -\beta )(1-\theta_{2})pD(1+i)\\ \;+(\alpha -\beta )(1-\theta_{2})pD(1+i)x] \end{array}$$

(22) $$J_{31}=-z(1-z)\{-\beta (F+\psi_{W2})-[(\alpha -\beta )(F+\psi_{W2})]y\}$$

(23) $$\begin{array}{l} J_{32}=z(1-z)[\psi_{R1}+\psi_{R2}+(\alpha -\beta )(F+\psi_{W2})-R_{L}\\ \;-(\alpha -\beta )(F+\psi_{W2})x] \end{array}$$

(24) $$\begin{array}{l} J_{33}=(1-2z)\{-\beta (F+\psi_{W2})x+[\psi_{R1}+\psi_{R2}\\ \;+(\alpha -\beta )(F+\psi_{W2})-R_{L}]y-[(\alpha -\beta )(F+\psi_{W2})]xy\\ \;+C_{R2}+\beta (F+\psi_{W2})-C_{R1}-M_{R}\} \end{array}$$

Substitute the eight pure strategy Nash equilibrium points in the Jacobian matrix (15), the corresponding eigenvalues can be obtained, and thus the asymptotic stability of the eight equilibrium points can be judged.

(1) For the equilibrium point *E*_1_(0, 0, 0), the eigenvalues of the Jacobian matrix are as follows:

(25) $$\{\begin{array}{ccc} \lambda_{1}^{1} & = & C_{P2}-C_{P1}-\theta_{1}pD\left(1+i\right)\\ \lambda_{2}^{1} & = & -C_{L}/D\;\\ \lambda_{3}^{1} & = & C_{R2}+\beta (F+\psi_{W2})-C_{R1}-M_{R} \end{array}$$

It is noted that $\lambda_{1}^{1}=C_{P2}-C_{P1}-\theta_{1}pD\left(1+i\right)<0$,$\lambda_{2}^{1}=-C_{L}/D<0$, thus, according to the judgment criterion, when *C*_*R*2_ + β(*F* + ψ_*W*2_) − *C*_*R*1_ − *M*_*R*_ < 0,*E*_1_(0, 0, 0) will be an ESS, in this case, the P2P lending platforms will adopt the “negative positive” strategy, the investors will adopt the “not participating in co-governance” strategy, and the financial regulators will adopt the “traditional regulation” strategy, which is the worst state.

(2) For the equilibrium point *E*_2_(1, 0, 0), the eigenvalues of the Jacobian matrix are as follows:

(26) $$\{\begin{array}{c} \lambda_{1}^{2}=-[C_{P2}-C_{P1}-\theta_{1}pD(1+i)]\\ \lambda_{2}^{2}=-C_{L}/D\;\\ \lambda_{3}^{2}=C_{R2}-C_{R1}-M_{R} \end{array}$$

According to the assumptions, $\lambda_{1}^{2}=-\left[C_{P2}-C_{P1}-\theta_{1}pD\left(1+i\right)\right]>0$, thus, the judgment criterion is not satisfied, and *E*_2_(1, 0, 0) cannot be an ESS.

(3) For the equilibrium point *E*_3_(0, 1, 0), the eigenvalues of the Jacobian matrix are as follows:

(27) $$\{\begin{array}{c} \lambda_{1}^{3}=\psi_{P1}+\psi_{P2}+C_{P2}-C_{P1}-\theta_{1}pD\left(1+i\right)\\ \lambda_{2}^{3}=C_{L}/D\\ \lambda_{3}^{3}=\psi_{R1}+\psi_{R2}+\alpha (F+\psi_{W2})-R_{L}+C_{R2}-C_{R1}-M_{R} \end{array}$$

where *C*_*L*_/*D* refers to the cost of investors when adopting the “participating in co-governance” strategy and cannot be negative, thus $\lambda_{2}^{3}>0$ does not meet the judgment criterion, and *E*_3_(0, 1, 0) cannot be an ESS.

(4) For the equilibrium point *E*_4_(0, 0, 1), the eigenvalues of the Jacobian matrix are as follows:

(28) $$\{\begin{array}{ccc} \lambda_{1}^{4} & = & \beta F+\beta \theta_{2}pD(1+i)+C_{P2}-C_{P1}-\theta_{1}pD(1+i)\\ \lambda_{2}^{4} & = & R_{L}-\frac{C_{L}}{D}-\left(\alpha -\beta \right)\left(1-\theta_{2}\right)pD\left(1+i\right)\\ \lambda_{3}^{4} & = & -\left[C_{R2}+\beta \left(F+\psi_{W2}\right)-C_{R1}-M_{R}\right] \end{array}$$

when β*F* + βθ_2_*pD*(1 + *i*) + *C*_*P*2_ − *C*_*P*1_ − θ_1_*pD*(1 + *i*) < 0,*R*_*L*_ − *C*_*L*_/*D* − (α − β)(1 − θ_2_)*pD*(1+*i*) < 0 and *C*_*R*2_ + β(*F* + ψ_*W*2_) − *C*_*R*1_ − *M*_*R*_ > 0, *E*_4_(0, 0, 1) will be an ESS.

(5) For the equilibrium point *E*_5_(0, 1, 1), the eigenvalues of the Jacobian matrix are as follows:

(29) $$\{\begin{array}{c} \lambda_{1}^{5}=\psi_{P1}+\psi_{P2}+\alpha F+\alpha \theta_{2}pD(1+i)+C_{P2}-C_{P1}-\theta_{1}pD(1+i)\\ \lambda_{2}^{5}=-\left[R_{L}-\frac{C_{L}}{D}-\left(\alpha -\beta \right)\left(1-\theta_{2}\right)pD\left(1+i\right)\right]\\ \lambda_{3}^{5}=-\left[\psi_{R1}+\psi_{R2}+\alpha \left(F+\psi_{W2}\right)-R_{L}+C_{R2}-C_{R1}-M_{R}\right] \end{array}$$

when ψ_*P*1_ + ψ_*P*2_ + α*F* + αθ_2_*pD*(1 + *i*) + *C*_*P*2_ − *C*_*P*1_ − θ_1_*pD*(1+*i*) < 0, *R*_*L*_−*C*_*L*_/*D*−(α−β) (1−θ_2_)*pD*(1+*i*) > 0 and ψ_*R*1_+ψ_*R*2_+α(*F*+ψ_*W*2_)−*R*_*L*_+*C*_*R*2_−*C*_*R*1_−*M*_*R*_ > 0,*E*_5_(0, 1, 1) will be an ESS.

(6) For the equilibrium point *E*_6_(1, 1, 0), the eigenvalues of the Jacobian matrix are as follows:

(30) $$\{\begin{array}{c} \lambda_{1}^{6}=-[\psi_{P1}+\psi_{P2}+C_{P2}-C_{P1}-\theta_{1}pD(1+i)]\\ \lambda_{2}^{6}=C_{L}/D\;\\ \lambda_{3}^{6}=\psi_{R1}+\psi_{R2}-R_{L}+C_{R2}-C_{R1}-M_{R} \end{array}$$

where *C*_*L*_refers to the cost of investors when adopting the “participating in co-governance” strategy and cannot be negative, thus $\lambda_{2}^{6}>0$ does not satisfy the judgment criterion, and *E*_6_(1, 1, 0) cannot be an ESS.

(7) For the equilibrium point *E*_7_(1, 0, 1), the eigenvalues of the Jacobian matrix are as follows:

(31) $$\{\begin{array}{c} \lambda_{1}^{7}=-[\beta F+\beta \theta_{2}pD(1+i)+C_{P2}-C_{P1}-\theta_{1}pD(1+i)]\\ \lambda_{2}^{7}=R_{L}-C_{L}/D\\ \lambda_{3}^{7}=-\left(C_{R2}-C_{R1}-M_{R}\right) \end{array}$$

Since $\lambda_{3}^{7}=-\left(C_{R2}-C_{R1}-M_{R}\right)>0$ does not satisfy the judgment criterion, thus, *E*_7_(1, 0, 1) cannot be an ESS.

(8) For the equilibrium point *E*_8_(1, 1, 1), the eigenvalues of the Jacobian matrix are as follows:

(32) $$\{\begin{array}{c} \lambda_{1}^{8}=-[\psi_{P1}+\psi_{P2}+\alpha F+\alpha \theta_{2}pD\left(1+i\right)\;\\ +C_{P2}-C_{P1}-\theta_{1}pD(1+i)]\\ \lambda_{2}^{8}=C_{L}/D-R_{L}\\ \lambda_{3}^{8}=-[\psi_{R1}+\psi_{R2}-R_{L}+C_{R2}-C_{R1}-M_{R}] \end{array}$$

When ψ_*P*1_+ψ_*P*2_+α*F*+αθ_2_*pD*(1+*i*)+*C*_*P*2_−*C*_*P*1_−θ_1_*pD*(1+*i*) > 0, *C*_*L*_/*D*−*R*_*L*_ < 0 and ψ_*R*1_+ψ_*R*2_−*R*_*L*_+*C*_*R*2_−*C*_*R*1_−*M*_*R*_ > 0, *E*_8_(1, 1, 1) will be an ESS.

Overall, the equilibrium points *E*_2_(1, 0, 0), *E*_3_(0, 1, 0), *E*_6_(1, 1, 0), and *E*_7_(1, 0, 1) cannot be ESSs under any conditions, while *E*_1_(0, 0, 0),*E*_4_(0, 0, 1),*E*_5_(0, 1, 1), and *E*_8_(1, 1, 1) can be ESSs under certain conditions. Based on the above analysis, the stability conditions of the four equilibrium points, which may be an ESS, are listed in Table 4.

**Table 4:** Stability conditions of the equilibrium points.

**Equilibrium point**	**Conditions of ESS**
*E*_1_(0, 0, 0)	*C*_*R*2_ + β(*F* + ψ_*W*2_) − *C*_*R*1_ − *M*_*R*_ < 0
*E*_4_(0, 0, 1)	β*F* + βθ_2_*pD*(1 + *i*) + *C*_*P*2_ − *C*_*P*1_ − θ_1_*pD*(1 + *i*) < 0,*R*_*L*_ − *C*_*L*_ − (α − β)(1 − θ_2_)*pD*(1 + *i*) < 0,
	*C*_*R*2_ + β(*F* + ψ_*W*2_) − *C*_*R*1_ − *M*_*R*_ > 0
*E*_5_(0, 1, 1)	ψ_*P*1_ + ψ_*P*2_ + α*F* + αθ_2_*pD*(1 + *i*) + *C*_*P*2_ − *C*_*P*1_ − θ_1_*pD*(1 + *i*) < 0,
	*R*_*L*_ − *C*_*L*_ − (α − β)(1 − θ_2_)*pD*(1 + *i*) > 0,
	ψ_*R*1_ + ψ_*R*2_ + α(*F* + ψ_*W*2_) − *R*_*L*_ + *C*_*R*2_ − *C*_*R*1_ − *M*_*R*_ > 0
*E*_8_(1, 1, 1)	ψ_*P*1_ + ψ_*P*2_ + α*F* + αθ_2_*pD*(1 + *i*) + *C*_*P*2_ − *C*_*P*1_ − θ_1_*pD*(1 + *i*) > 0,
	*C*_*L*_ − *R*_*L*_ < 0,
	ψ_*R*1_ + ψ_*R*2_ − *R*_*L*_ + *C*_*R*2_ − *C*_*R*1_ − *M*_*R*_ > 0

Comparing the four possible ESSs, from the perspective of consumer protection and the sustainable development of the FinTech industry, *E*_8_(1, 1, 1) is the most appropriate ESS, i.e., P2P lending platforms adopt the “positive disposal” strategy, investors adopt the “participating in co-governance” strategy, and financial regulators adopt the “co-governance regulation” strategy. The reasons are as follows: First, P2P lending platforms will be eager to adopt a “positive disposal” strategy, as this will improve their reputation and establish a solid foundation for the future development of the FinTech industry. Second, the fundamental purpose of the co-governance regulation is to protect the consumer, only when the investors adopt a “participating in co-governance” strategy, do they have the possibility to take their money back quickly and smoothly. Third, the current benign exit of P2P lending platforms in China is inseparable from the regulation of the financial regulators, if the investors can cooperate with the financial regulators to form a social co-governance pattern with high regulatory efficiency, then the benign exit of P2P lending platforms will be accelerated, and the rights and interests of the consumer will be ensured.

To guarantee that *E*_8_(1, 1, 1) is the only evolutionary stable strategy of the tripartite game, the stability constraints are listed as follows:

(1) $\lambda_{1}^{8}<0$, $\lambda_{2}^{8}<0$ and $\lambda_{3}^{8}<0$. Correspondingly, ψ_*P*1_ + ψ_*P*2_ + α*F* + αθ_2_*pD*(1 + *i*) + *C*_*P*2_ − *C*_*P*1_ − θ_1_*pD*(1 + *i*) > 0 means that for P2P lending platforms the payoffs of adopting a “positive disposal” strategy with reputation benefits should be higher than that of adopting a “negative disposal” strategy after suffering a reputation loss and being punished by the financial regulators. *C*_*L*_/*D*−*R*_*L*_ < 0 means that for investors the rewards of adopting the “participating in co-governance” strategy should be higher than the costs. ψ_*R*1_ + ψ_*R*2_ − *R*_*L*_ + *C*_*R*2_ − *C*_*R*1_ − *M*_*R*_ > 0 means that for financial regulators, the payoff difference between co-governance regulation and traditional regulation should be higher than the sum of the initiative cost to set up the social co-governance pattern and the rewards paid to the investors. The three conditions need to be satisfied simultaneously.
(2) $\lambda_{3}^{1}>0$. Correspondingly, *C*_*R*2_+β(*F*+ψ_*W*2_)−*C*_*R*1_−*M*_*R*_ > 0 means that for financial regulators the cost of adopting “traditional regulation” is higher than that of adopting “co-governance regulation.”
(3) $\lambda_{1}^{4}>0$, or $\lambda_{2}^{4}>0$. Correspondingly, β*F* + βθ_2_*pD*(1 + *i*) + *C*_*P*2_ − *C*_*P*1_ − θ_1_*pD*(1 + *i*) > 0 means that for P2P lending platforms the cost of adopting aa “negative disposal” strategy after being punished by the financial regulators should of higher than that of adopting a “positive disposal” strategy. *R*_*L*_−*C*_*L*_/*D*−(α−β)(1−θ_2_)*pD*(1+*i*) > 0 means that for investors the payoffs of “participating in co-governance” should be higher than that of “not participating in co-governance.” However, since $\lambda_{3}^{4}=-\lambda_{3}^{1}$, thus, when condition (2) holds, $\lambda_{3}^{4}\;$ cannot be positive.
(4) $\lambda_{1}^{5}>0$, or $\lambda_{2}^{5}>0$, or $\lambda_{3}^{5}>0$. From the above analysis, $\lambda_{1}^{5}=-\lambda_{1}^{8}$, thus, when condition (1) is satisfied, then condition (2) holds.

To guarantee that *E*_8_(1, 1, 1) is the only evolutionary stable strategy of the tripartite game, the above four conditions must be held at the same time.

## Numerical simulation

Through the theoretical analysis illustrated above, four ESSs have been identified, which can be obtained when corresponding conditions are satisfied. Moreover, to intuitively observe the evolutionary trajectories of the stakeholders and their sensitivity to parameters, it is necessary to simulate their strategies. In this study, we implemented this simulation by using MATLAB.

### Evolutionary stable strategies

#### Scenario 1

**Figure 3:**
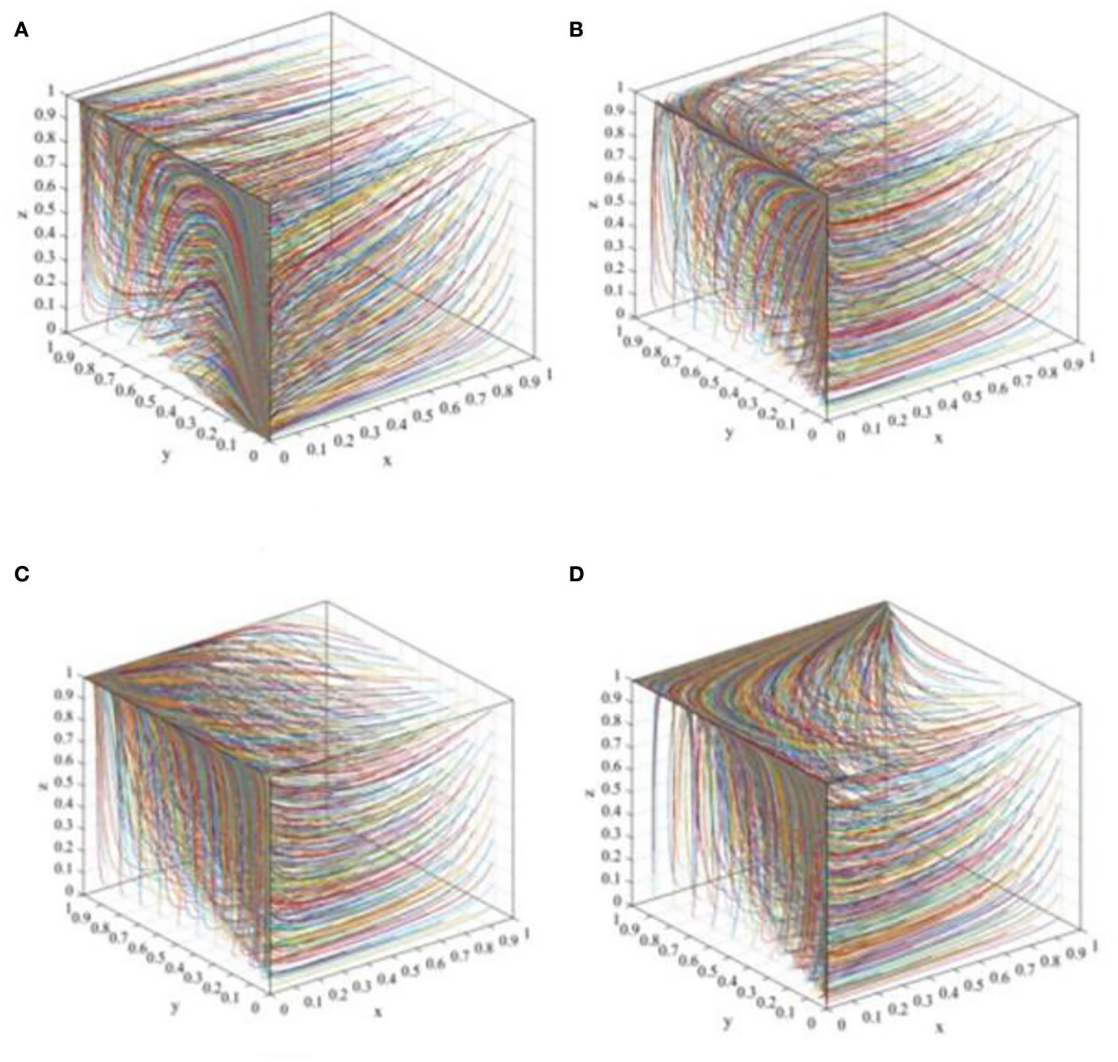
The evolutionary path diagram of the three participants in different scenarios. **(A)** The evolutionary path at the equilibrium point *E*_1_(0, 0, 0); **(B)** The evolutionary path at the equilibrium point *E*_4_(0, 0, 1); **(C)** The evolutionary path at the equilibrium point *E*_5_(0, 1, 1); and **(D)** The evolutionary path at the equilibrium point *E*_8_(1, 1, 1).

In this scenario, when *C*_*R*2_ + β(*F* + ψ_*W*2_)−*C*_*R*1_ − *M*_*R*_ < 0, *E*_1_(0, 0, 0) will be the ESS. In order to meet these conditions, suppose that *C*_*P*1_ = 3, *C*_*P*2_ = 1, θ_1_= 0.9, θ_2_ = 0.2, *F* = 1, ψ_*P*1_ = 0.5, ψ_*P*2_ = 1, *R*_*L*_ = 0.5, *C*_*L*_ = 0.5, *D* = 0.5, *P* = 10, *i* = 0.1, *C*_*R*1_ = 5.5, *C*_*R*2_ = 3.5, α = 0.8, β = 0.5, ψ_*w*1_ = 4, ψ_*w*2_ = 6, ψ_*R*1_ = 2, ψ_*R*2_ = 4 and *M*_*R*_ = 5. Then, as shown in Figure 3A, regardless of the initial proportion of the three participants, the definitive evolutionary result is *E*_1_(0, 0, 0). The main reason for this result is that the payoff of P2P lending platforms' “negative disposal” strategy is higher than that of “positive disposal” due to the limited penalty of financial regulators to the P2P lending platforms and the lack of reputation effect, while the payoff of investors participating in co-governance is lower than their basic earning, and the reputation benefits of the financial regulators are not enough to offset the difference between the fine and co-governance regulation cost. Thus, the ESS of P2P lending platforms, investors, and financial regulators is “negative disposal,” “not participating in co-governance,” and “traditional regulation,” respectively.

#### Scenario 2

In this scenario, when β*F* + βθ_2_*pD*(1 + *i*) + *C*_*P*2_ − *C*_*P*1_ − θ_1_*pD*(1 + *i*) < 0, *R*_*L*_ − *C*_*L*_/*D* − (α − β)(1 − θ_2_)*pD*(1 + *i*) < 0 and *C*_*R*2_ + β(*F* + ψ_*W*2_) − *C*_*R*1_ − *M*_*R*_ > 0, the equilibrium point *E*_4_(0, 0, 1) will be the ESS. To meet these conditions, suppose that *C*_*P*1_ = 3,*C*_*P*2_ = 1,θ_1_=0.9, θ_2_ = 0.2, *F* = 4, ψ_*P*1_ = 0.5, ψ_*P*2_ = 1, *R*_*L*_ = 1, *C*_*L*_ = 0.5, *D* = 0.5, *P* = 10, *i* = 0.1, *C*_*R*1_ = 5.5, *C*_*R*2_ = 3.5, α = 0.8, β = 0.5, ψ_*w*1_ = 4, ψ_*w*2_ = 6, ψ_*R*1_ = 3, ψ_*R*2_ = 5 and *M*_*R*_ = 2. Then, Figure 3B shows that the proportion of P2P lending platforms adopting the “positive disposal” strategy, and the proportion of investors adopting the “not participating in co-governance” strategy constantly decreases with the iteration of the evolution, and eventually converges to 0, whereas the proportion of financial regulators adopting “co-governance regulation” continually increases, and eventually converges to 1. Clearly, the reasons why P2P lending platforms adopt such a strategy is similar to scenario 1, and since the rewards of financial regulators to investors are not attractive to offset the costs, then the investors will still adopt the “not participating in co-governance” strategy. However, the payoff of financial regulators' “co-governance regulation” is higher than that of the “traditional regulation” strategy; thus, in this scenario, financial regulators are more willing to adopt the “co-governance regulation” strategy.

#### Scenario 3

In this scenario, when ψ_*P*1_+ψ_*P*2_+α*F*+αθ_2_*pD*(1+*i*)+*C*_*P*2_−*C*_*P*1_−θ_1_*pD*(1+*i*) < 0, *R*_*L*_−*C*_*L*_/*D*−(α−β)(1−θ_2_)*pD*(1+*i*) > 0 and ψ_*R*1_ + ψ_*R*2_ + α(*F* + ψ_*W*2_) − *R*_*L*_ + *C*_*R*2_ − *C*_*R*1_ − *M*_*R*_ > 0, the equilibrium point *E*_5_(0, 1, 1) is the ESS. To meet these conditions, suppose that *C*_*P*1_ = 3, *C*_*P*2_ = 1, θ_1_=0.9, θ_2_ = 0.2, *F* = 5, ψ_*P*1_ = 0.5, ψ_*P*2_ = 1, *R*_*L*_ = 3, *C*_*L*_ = 0.5, *D* = 0.5, *P* = 10, *i* = 0.1, *C*_*R*1_ = 5.5, *C*_*R*2_ = 3.5, α = 0.8, β = 0.5, ψ_*w*1_ = 4, ψ_*w*2_ = 6, ψ_*R*1_ = 3, ψ_*R*2_ = 5 and *M*_*R*_ = 2. Then, Figure 3C shows that the ESS will eventually stabilize at the equilibrium point *E*_5_(0, 1, 1). This phenomenon demonstrates that the financial regulators will adopt the “co-governance regulation” strategy due to the increase in their payoffs, and the investors will also adopt the “co-governance” strategy due to the increase in rewards. However, since there does not exist solid reputation mechanism for the P2P lending platforms and the penalty is not properly formulated, thus, after comparing the payoffs of the “positive disposal” and “negative disposal” strategy, the P2P lending platforms will eventually adopt the “negative disposal” strategy.

#### Scenario 4

In this scenario, when ψ_*P*1_ + ψ_*P*2_ + α*F* + αθ_2_*pD*(1 + *i*) + *C*_*P*2_ − *C*_*P*1_ − θ_1_*pD*(1 + *i*) > 0,*C*_*L*_/*D* − *R*_*L*_ < 0 and ψ_*R*1_ + ψ_*R*2_ − *R*_*L*_ + *C*_*R*2_ − *C*_*R*1_ − *M*_*R*_ > 0, the equilibrium point *E*_8_(1, 1, 1) is the ESS. To meet these conditions, suppose that *C*_*P*1_ = 3, *C*_*P*2_ = 1, θ_1_=0.9, θ_2_ = 0.2, *F* = 5, ψ_*P*1_ = 2, ψ_*P*2_ = 3, *R*_*L*_ = 3, *C*_*L*_ = 0.5, *D* = 0.5, *P*=10, *i* = 0.1, *C*_*R*1_ = 5.5, *C*_*R*2_ = 3.5, α = 0.8, β = 0.5, ψ_*w*1_ = 4, ψ_*w*2_ = 6, ψ_*R*1_ = 3, ψ_*R*2_ = 5 and *M*_*R*_ = 2. Then, as shown in Figure 3D, the proportion of P2P lending platforms adopting the “positive disposal” strategy, the proportion of investors adopting the “not participating in co-governance” strategy, and the proportion of financial regulators adopting “co-governance regulation” continually increase and eventually converges to 1. In this case, the financial regulators are more willing to carry out co-governance regulation due to the high reputation benefits from the investors and the decrease in the initiative cost to set up the social co-governance pattern. Accordingly, the investors will adopt the “co-governance” strategy due to the high rewards. Consequently, if the P2P lending platforms adopt the “negative disposal” strategy, then they need not only to pay corresponding fines but also suffer a great loss of their reputation, which is fatal to their future development; thus, the P2P lending platforms will eventually adopt the “positive disposal” strategy.

### Sensitivity analysis

From the above analysis, the equilibrium point *E*_8_(1, 1, 1) is the ideal ESS of the tripartite evolutionary model, in order to more intuitively illustrate the influence of the changes in parameters on the stable state of the tripartite evolutionary game, this subsection will use numerical simulations to explore the sensitivity of the three participants to some key parameters, i.e., the initial proportion of three participants, the cost of P2P lending platforms when adopting “positive disposal” strategy (*C*_*P*1_), the penalty of financial regulators to P2P lending platforms (*F*), the reputation effects of P2P lending platforms (ψ_*P*1_,ψ_*P*2_), the rewards of financial regulators to investors (*R*_*L*_), the cost of investors (*C*_*L*_), the centrality degree (*D*), the investment volume of investors (*P*), the reputation benefits of financial regulators (ψ_*R*1_), and the initiative cost of financial regulators to set up the co-governance regulation pattern (*M*_*R*_). It should be noted that when we analyze the sensitivity of one of these parameters, the remaining parameters' values remain unchanged. The initial settings of the parameters are the same as in scenario 4.

#### The initial proportion of three participants

To further illustrate the influence of the initial proportion of three participants on the tripartite evolutionary game, we let *x*_0_ equals 0.3, 0.5, and 0.7, *y*_0_ equals 0.3, 0.5, and 0.7, *z*_0_ also equals 0.3, 0.5, and 0.7 in turn. It is noteworthy that the initial states of the remaining stakeholders are controlled at 0.5 when we probe the sensitivity of each stakeholder to the initial state.

**Figure 4:**
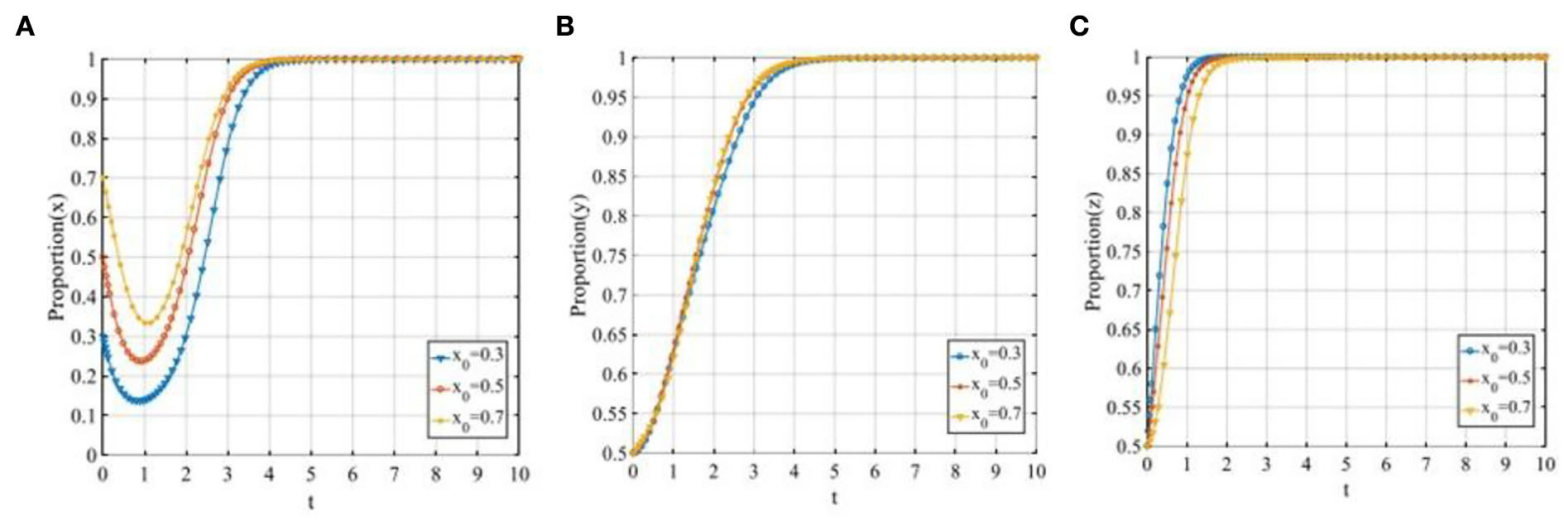
The evolutionary trajectories of three participants when *x*_0_ = 0.3, 0.5, 0.7. **(A)** The sensitivity of P2P lending platforms; **(B)** The sensitivity of investors; and **(C)** The sensitivity of financial regulators.

Set *x*_0_ = 0.3, 0.5, 0.7, the evolutionary trajectories of the three participants are shown in Figure 4. The change in the initial proportion of P2P lending platforms has a significant impact on P2P lending platforms, the higher the initial proportion is, the faster the P2P lending platforms will converge to 1. While, as shown in Figures 4B,C, the change in the initial proportion of P2P lending platforms has a limited effect on investors and financial regulators.

**Figure 5:**
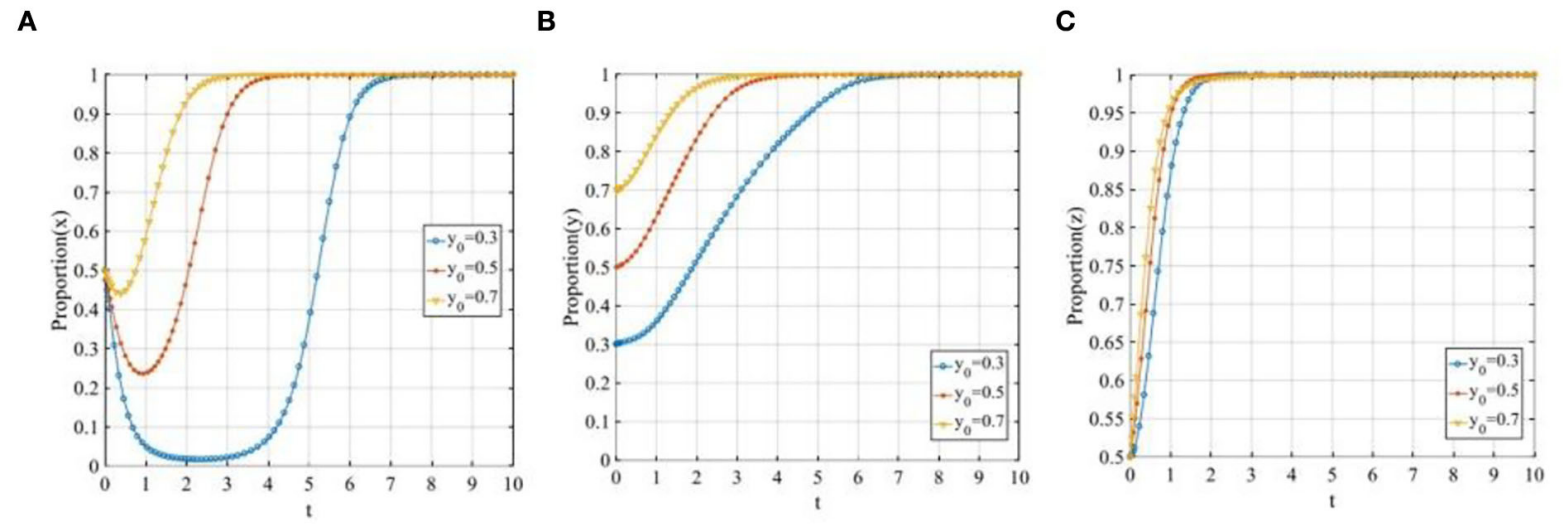
The evolutionary trajectories of three participants when *y*_0_ = 0.3, 0.5, 0.7. **(A)** The sensitivity of P2P lending platforms; **(B)** The sensitivity of investors; and **(C)** The sensitivity of financial regulators.

Set *y*_0_ = 0.3, 0.5, 0.7, the evolution of the three participants is shown in Figure 5. The change in the initial proportion of investors has a significant impact on P2P lending platforms and investors while having a limited effect on financial regulators. With the increase in the initial proportion of the investors adopting the “participating in co-governance” strategy, the convergence rates of the P2P lending platforms and investors will also increase, the higher the initial proportion is, the faster the P2P lending platforms and investors will converge to 1.

**Figure 6:**
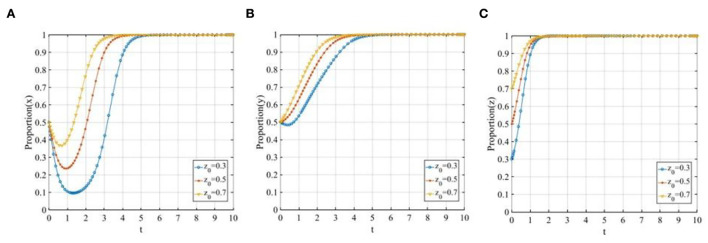
The evolutionary trajectories of three participants when *z*_0_ = 0.3, 0.5, 0.7. **(A)** The sensitivity of P2P lending platforms; **(B)** The sensitivity of investors; and **(C)** The sensitivity of financial regulators.

Set *z*_0_ = 0.3, 0.5, 0.7, the evolutionary trajectories of the three participants are shown in Figure 6. The change in the initial proportion of financial regulators has a significant impact on P2P lending platforms, investors, and financial regulators, respectively. With the increase in the initial proportion of the financial regulators adopting the “co-governance regulation” strategy, the convergence rates of the three participants will all increase, the higher the initial proportion is, the faster the evolutionary trajectories will finally stabilize at the ideal state *E*_8_(1, 1, 1).

From the above analysis, we can see that the initial proportion of the three participants will impact the speed to reach the final ESS but would not change the final stable state. Therefore, we set *x*_0_ = *y*_0_ = *z*_0_ = 0.5 to further illustrate the sensitivity of other parameters in the following sections.

#### The cost of P2P lending platforms' “positive disposal” strategy

**Figure 7:**
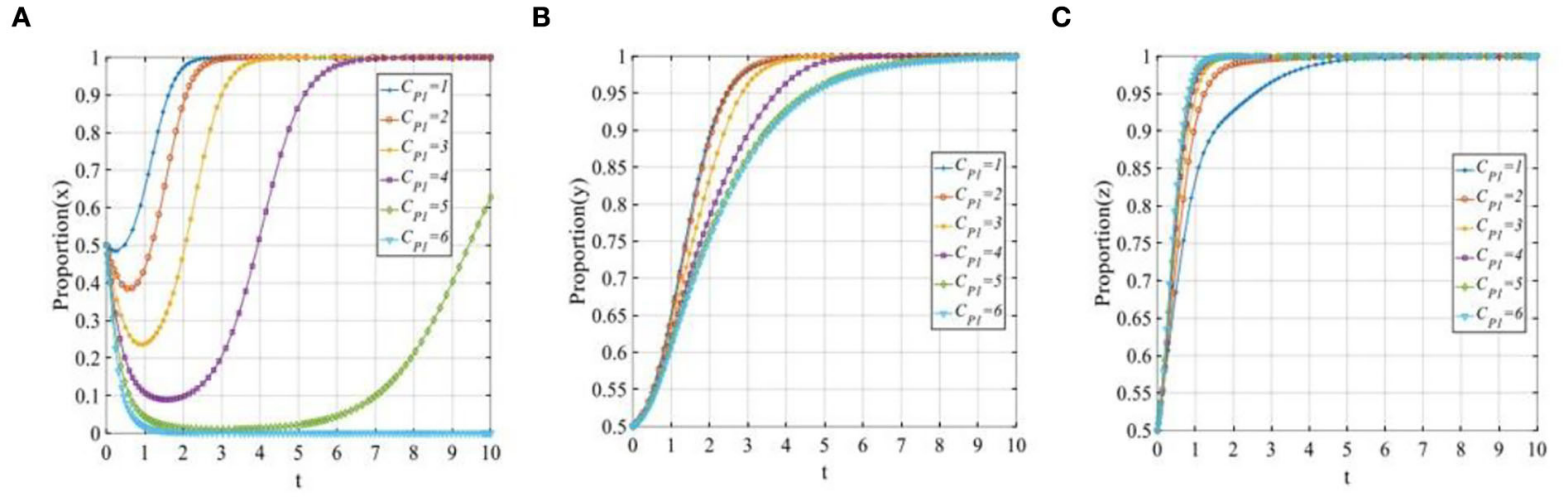
The sensitivity analysis of the cost of P2P lending platforms' “positive disposal” strategy when *C*_*P*1_ = 1, 2, 3, 4, 5, 6. **(A)** The sensitivity of P2P lending platforms; **(B)** The sensitivity of investors; and **(C)** The sensitivity of financial regulators.

To explore the sensitivity of three participants to the cost of P2P lending platforms when adopting a “positive disposal” strategy, let *C*_*P*1_ equals 1, 2, 3, 4, 5, and 6, respectively. The evolutionary trajectories of the three participants are shown in Figure 7. As seen from Figure 7A, P2P lending platforms are more willing to adopt the “positive disposal” strategy when the costs are relatively low. However, with the increase in costs, the evolutionary speed of the P2P lending platforms will be slower, meanwhile, once the costs exceed the threshold, P2P lending platforms will change their strategy from 1 to 0. Figure 7B shows that with the increase in costs, although investors will eventually adopt the “participating in co-governance” strategy, the time to reach the stable status will be continually prolonged. Figure 7C shows that with the increase in costs, the evolutionary speed of financial regulators will be accelerated.

#### The penalty from financial regulators for P2P lending platforms

**Figure 8:**
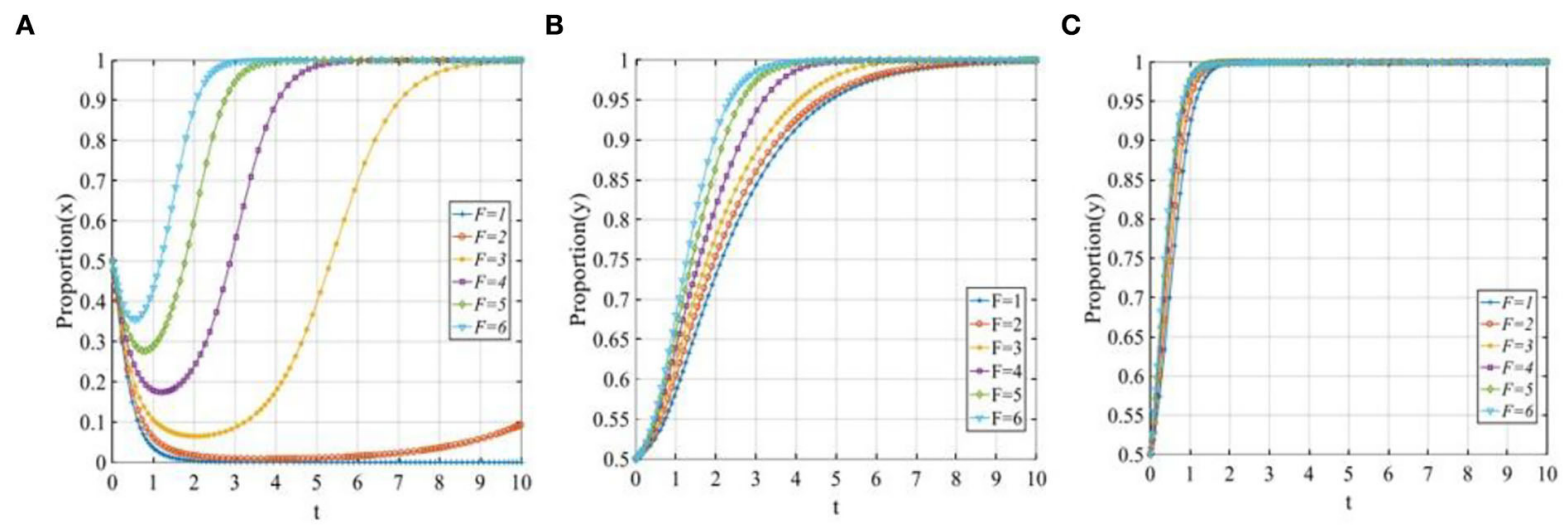
The sensitivity analysis of the penalty of financial regulators to P2P lending platforms when *F* = 1, 2, 3, 4, 5, 6. **(A)** The sensitivity of P2P lending platforms; **(B)** The sensitivity of investors; and **(C)** The sensitivity of financial regulators.

To explore the sensitivity of three participants to the penalty of financial regulators to P2P lending platforms, let *F* equals to 1, 2, 3, 4, 5, and 6. The evolutionary trajectories of the three participants are shown in Figure 8. As seen from Figure 8A, it is obvious that if the penalty is not properly formulated, the P2P lending platforms will eventually adopt the “negative disposal” strategy, when the penalty increases gradually, the P2P lending platforms will tend to adopt the “positive disposal” strategy, and with the increase in the penalty, the evolutionary speed will be improved sharply. Figure 8B shows that with the increase in the penalty, the evolutionary speed of the investors adopting “participating in co-governance” will be accelerated. Figure 8C shows that the evolutionary speed of the financial regulators will also be accelerated, but the effect is not significant compared with the other two participants.

#### The reputation effects of P2P lending platforms

**Figure 9:**
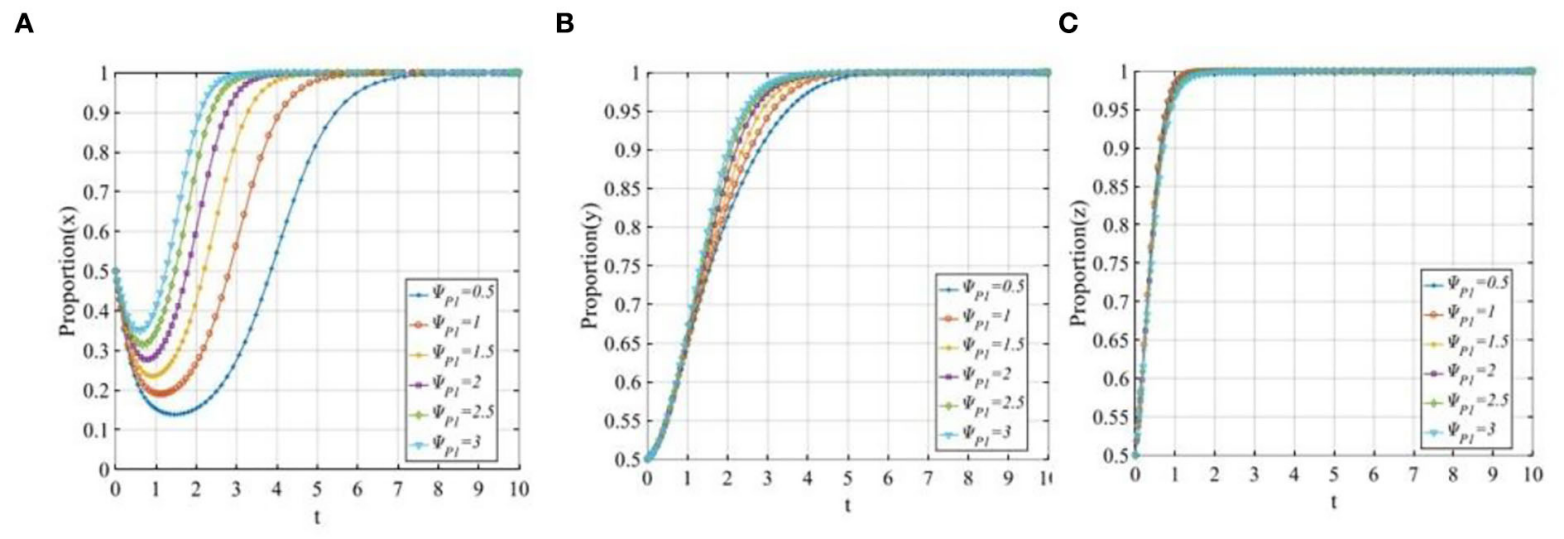
The sensitivity analysis of the reputation benefits of P2P lending platforms when ψ_*P*1_ = 0.5, 1, 1.5, 2, 2.5, 3. **(A)** The sensitivity of P2P lending platforms; **(B)** The sensitivity of investors; and **(C)** The sensitivity of financial regulators.

To explore the sensitivity of three participants to the reputation benefits of P2P lending platforms, let ψ_*P*1_ equals 0.5, 1, 1.5, 2, 2.5, and 3. The evolutionary trajectories of the three participants are shown in Figure 9. With the increase of the reputation benefits of the P2P lending platforms adopting a “positive disposal” strategy, the convergence rates of the P2P lending platforms and investors will also increase, the higher the reputation benefit is, the faster the P2P lending platforms and investors will converge to 1. However, in Figure 9C, the nearly overlapping curves demonstrate that the penalty has little effect on the financial regulators.

**Figure 10:**
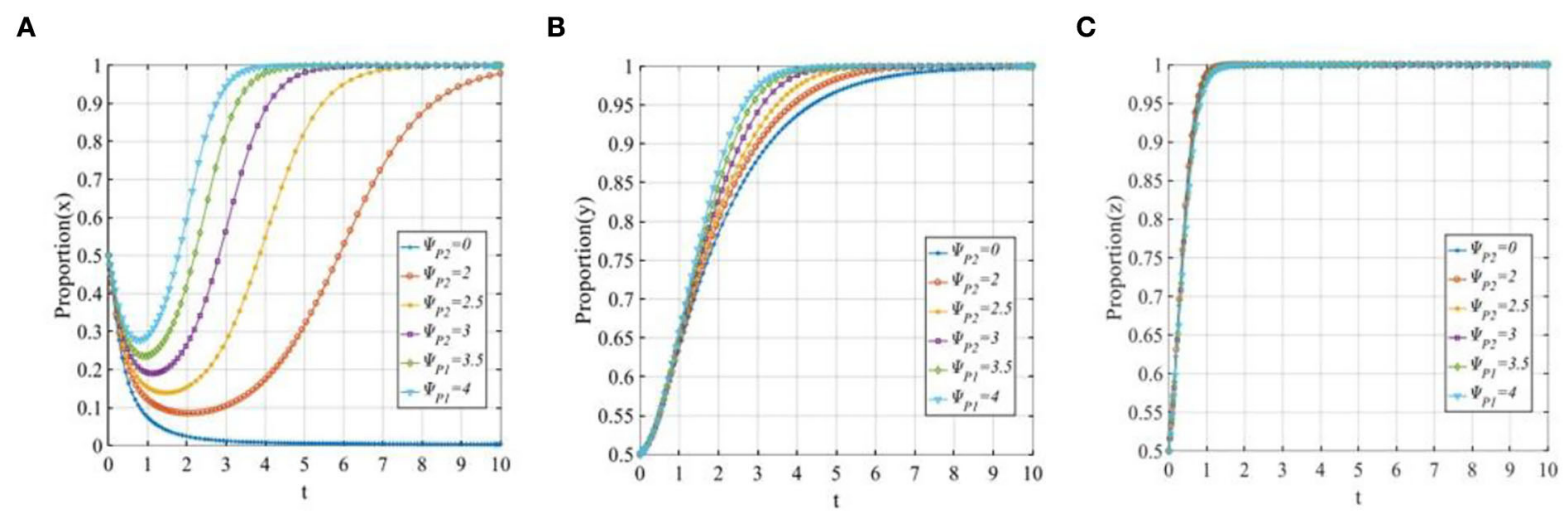
The sensitivity analysis of the reputation losses of P2P lending platforms when ψ_*P*2_ = 0, 2, 2.5, 3, 3.5, 4. **(A)** The sensitivity of P2P lending platforms; **(B)** The sensitivity of investors; and **(C)** The sensitivity of financial regulators.

To explore the sensitivity of three participants to the reputation loss of P2P lending platforms, let ψ_*P*2_ equals 0, 2, 2.5, 3, 3.5, and 4. The evolutionary trajectories of the three participants are shown in Figure 10. As seen from Figure 10A, P2P lending platforms tend to adopt the “positive disposal” strategy except for a sudden change in reputation loss (ψ_*P*2_ = 0). This means that when the reputation losses exceed the threshold, the P2P lending platforms will adopt the “positive disposal” strategy, and the evolutionary speed will be improved with the increase in the reputation losses. However, if the reputation losses of P2P lending platforms adopting a “negative disposal” strategy are lower than the threshold, then the P2P lending platforms will change their strategy from 1 to 0. Figure 10B shows that with the continual increase in reputation losses, the time of the investors adopting the “participating in co-governance” strategy will be continually shortened. In Figure 10C, similarly to the case of reputation benefits, reputation losses have little significant effect on the financial regulators.

#### The rewards of financial regulators to investors

**Figure 11:**
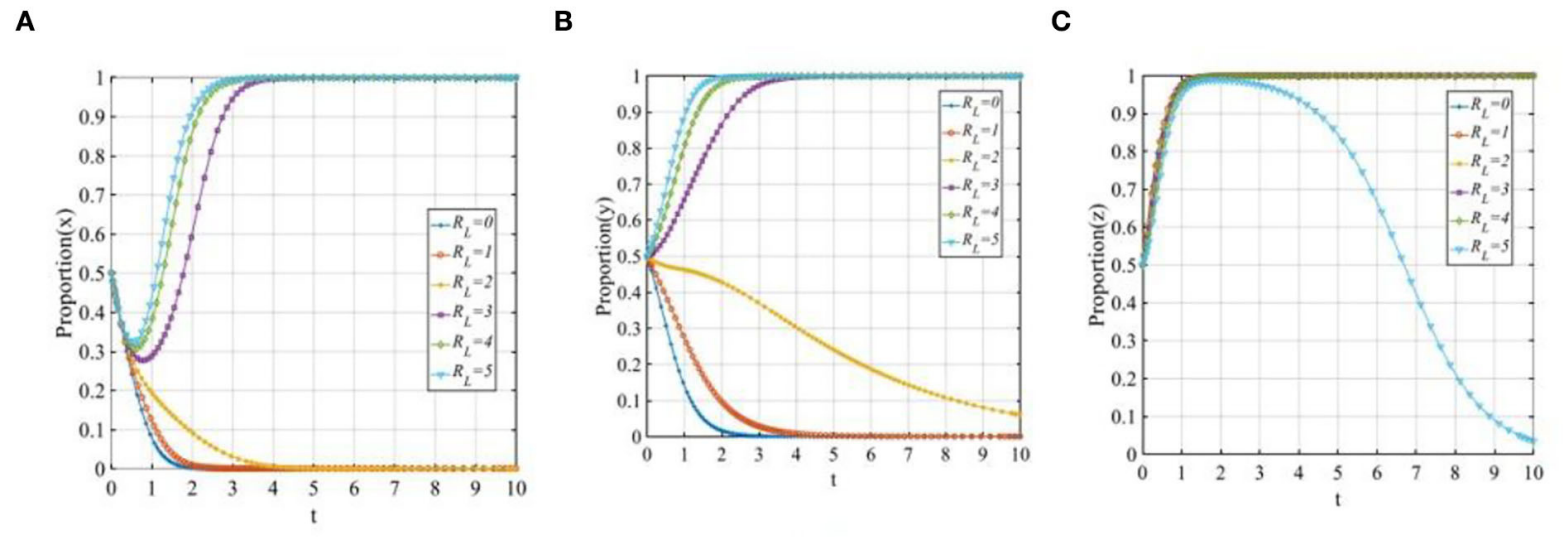
The sensitivity analysis of the rewards of financial regulators to investors when *R*_*L*_ = 0, 1, 2, 3, 4, 5. **(A)** The sensitivity of P2P lending platforms; **(B)** The sensitivity of investors; and **(C)** The sensitivity of financial regulators.

To explore the sensitivity of three participants to the rewards of financial regulators to investors, let *R*_*L*_ equals 0, 1, 2, 3, 4, and 5. The evolutionary trajectories of the three participants are shown in Figure 11. The change in the rewards of financial regulators to investors has a significant impact on P2P lending platforms, investors, and financial regulators, respectively. Figure 11A shows that with the increase in the rewards, the P2P lending platforms will change their strategy from 0 to 1. Figure 11B shows that if the rewards are small, the investor is not willing to adopt the “participating in co-governance” strategy, once the rewards exceed the threshold, the investors will tend to adopt the “participating in co-governance” strategy, and the evolutionary time will be shorted with the increase in the rewards. Interestingly, Figure 11C shows that the sensitivity of the financial regulators to the rewards is opposite to those of the P2P lending platforms and the investors, once the rewards exceed the threshold, the financial regulators will tend to adopt the “traditional regulation” strategy.

#### The cost to investors

**Figure 12:**
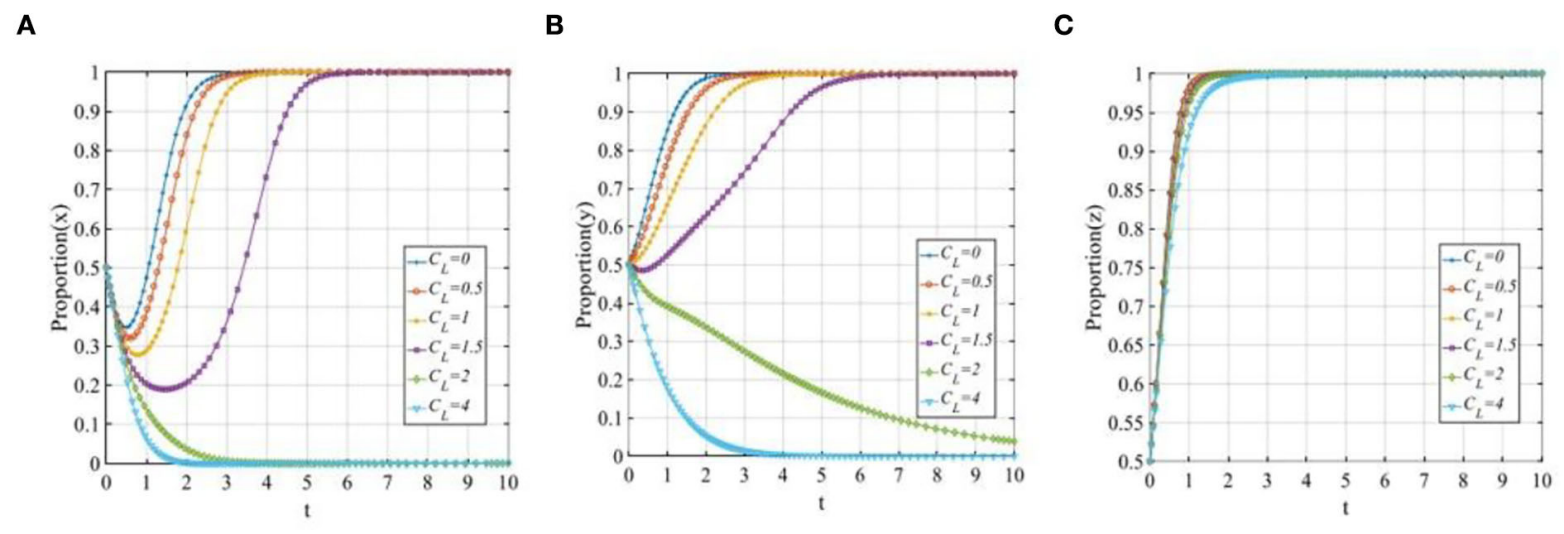
The sensitivity analysis of the cost of investors when *C*_*L*_ = 0, 0.5, 1, 1.4, 2, 4. **(A)** The sensitivity of P2P lending platforms; **(B)** The sensitivity of investors; **(C)** The sensitivity of financial regulators.

To explore the sensitivity of three participants to the cost of investors when adopting the “participating in co-governance” strategy, let *C*_*L*_ equals 0, 0.5, 1, 1.5, 2, and 4. The evolutionary trajectories of the three participants are shown in Figure 12. With the increase in the cost of the investors adopting the “participating in co-governance” strategy, the P2P lending platforms and investors will change their strategy from 1 to 0, and the higher the costs of investors are, the faster the P2P lending platforms and investors will converge to 0. Additionally, Figure 12C shows that with the increase in the costs of investors, although financial regulators will eventually choose the “co-governance regulation” strategy, the time to reach a stable status will be continually prolonged.

#### The centrality degree of investors

**Figure 13:**
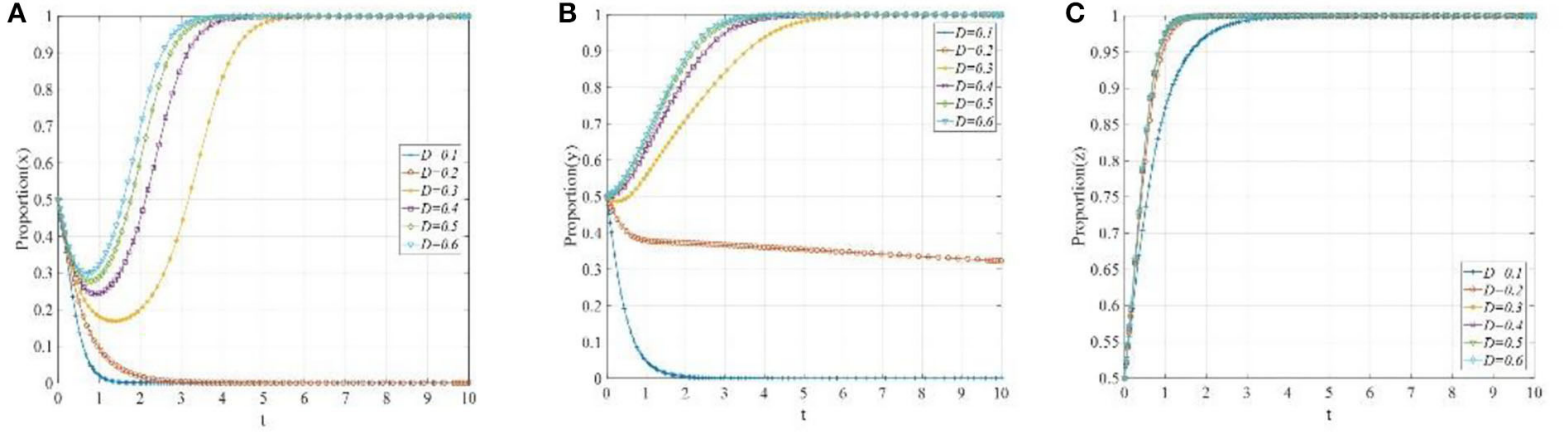
The sensitivity analysis of the centrality degree of investors when *D* = 0.1, 0.2, 0.3, 0.4, 0.5, 0.6. **(A)** The sensitivity of P2P lending platforms; **(B)** The sensitivity of investors; and **(C)** The sensitivity of financial regulators.

Network centrality plays a critical role in shaping lenders' investment behavior. According to the prior study (Chen et al., 2022), lenders who are in the center of a network not only invest by larger amounts but also more swiftly than their peers, reflecting the experience and information advantage arising from their position in the network. To explore the sensitivity of three participants toward the network centrality of investors, we use the centrality degree *D* (ranges from 0 to 1) (Freeman, 1978) to measure the network centrality of investors (Chen et al., 2022). We present sensitivity analysis for *D* equals 0.1, 0.2, 0.3, 0.4, 0.5, and 0.6, respectively. The evolutionary trajectories of the three participants are shown in Figure 13. From Figure 13A, we can see that with the increase in the centrality degree of investors (invest by higher fractions and more swiftly), the P2P lending platforms will change their strategy from 0 to 1. Similarly, Figure 13B shows that if the centrality degree of investors is low, the investors are not willing to adopt the “participating in co-governance” strategy. However, when the centrality degree of investors exceeds the threshold (*D* = 0.2), the investors would tend to adopt the “participating in co-governance” strategy, and the evolutionary time would be reduced with the increase in the centrality degree of investors. Figure 13C shows that the financial regulators' evolutionary speed to adopt the “participating in co-governance” strategy will also be accelerated as the centrality degree of investors increases, but this effect is not significant compared with the other two players.

#### The investment volume of investors

**Figure 14:**
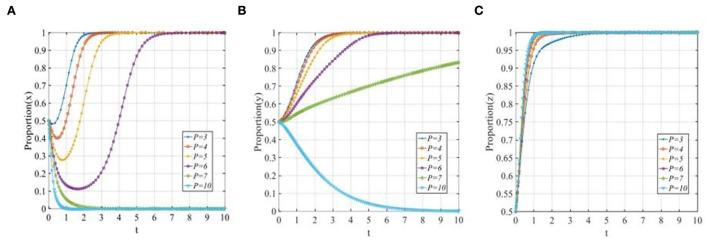
The sensitivity analysis of the investment volume of investors when *P* = 3, 4, 5, 6, 7, 10. **(A)** The sensitivity of P2P lending platforms; **(B)** The sensitivity of investors; and **(C)** The sensitivity of financial regulators.

To explore the sensitivity of three participants to the investment volume of investors, let *P* equal 3, 4, 5, 6, 7, and 10. The evolutionary trajectories of the three participants are shown in Figure 14. As seen from Figure 14A, P2P lending platforms are more willing to adopt the “positive disposal” strategy when the invest volumes are relatively low. However, when the invest volume increases gradually and exceeds the threshold, P2P lending platforms will change their strategy from 1 to 0. Similarly, Figure 14B shows that with the increase in the invest volume, there also exists a threshold for the investors, once the invest volume exceeds the threshold, the investors will change their strategies from 1 to 0. Figure 14C shows that with the increase in the invest volume, the evolutionary time of the financial regulators adopting the “co-governance regulation” strategy will be prolonged.

#### The initial cost of financial regulators to set up the co-governance regulation pattern

**Figure 15:**
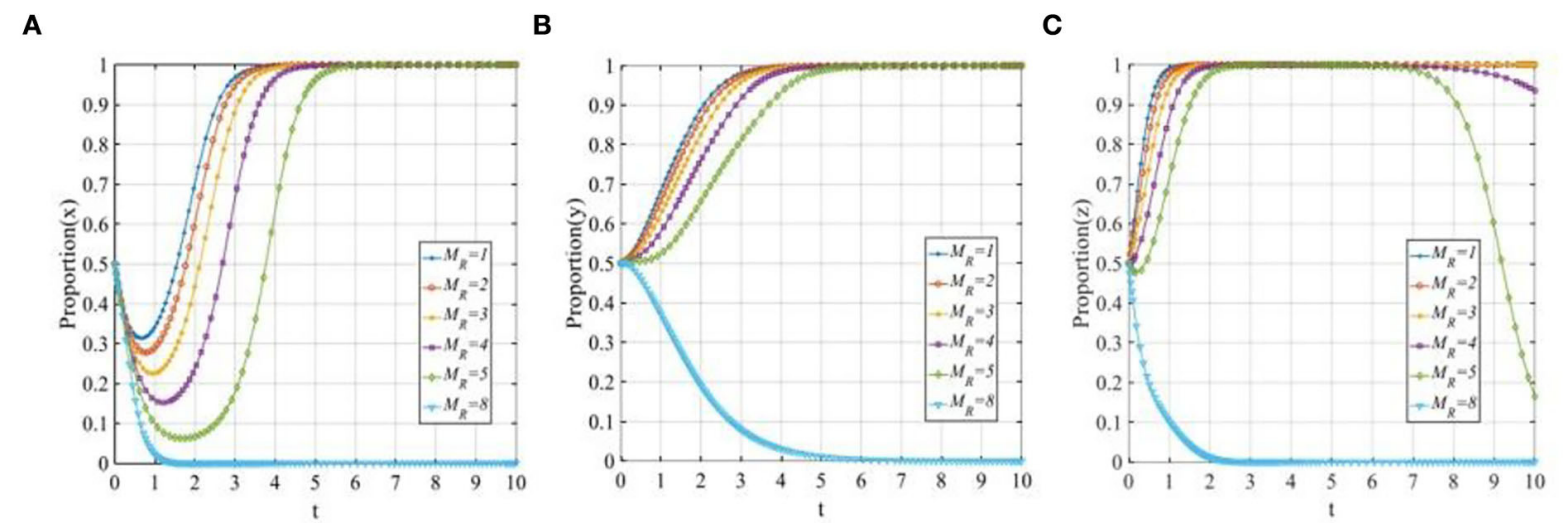
The sensitivity analysis of the initial cost of financial regulators to set up the co-governance regulation pattern when *M*_*R*_ = 1, 2, 3, 4, 5, 8. **(A)** The sensitivity of P2P lending platforms; **(B)** The sensitivity of investors; and **(C)** The sensitivity of financial regulators.

To explore the sensitivity of three participants to the initiative cost of financial regulators to set up the co-governance regulation pattern, let *M*_*R*_ equals 1, 2, 3, 4, 5, and 8. The evolutionary trajectories of the three participants are shown in Figure 15. When the initiative cost of financial regulators to set up the co-governance regulation pattern is low, the P2P lending platforms, the investors, and the financial regulators will adopt the “positive disposal,” “participating in co-governance,” and “co-governance regulation” strategy, respectively. However, with the increase in the initial cost of financial regulators, their evolutionary time will be prolonged, once the initial cost of financial regulators is too high, i.e., *M*_*R*_ = 8, then all three participants will change their strategy from 1 to 0.

## Discussion

### Implications

From the theoretical analysis and numerical simulation of the tripartite evolutionary model, we find that several variables play important roles to enhance the final evolutionary stable state with the implementation of the social co-governance pattern.

**Figure 16:**
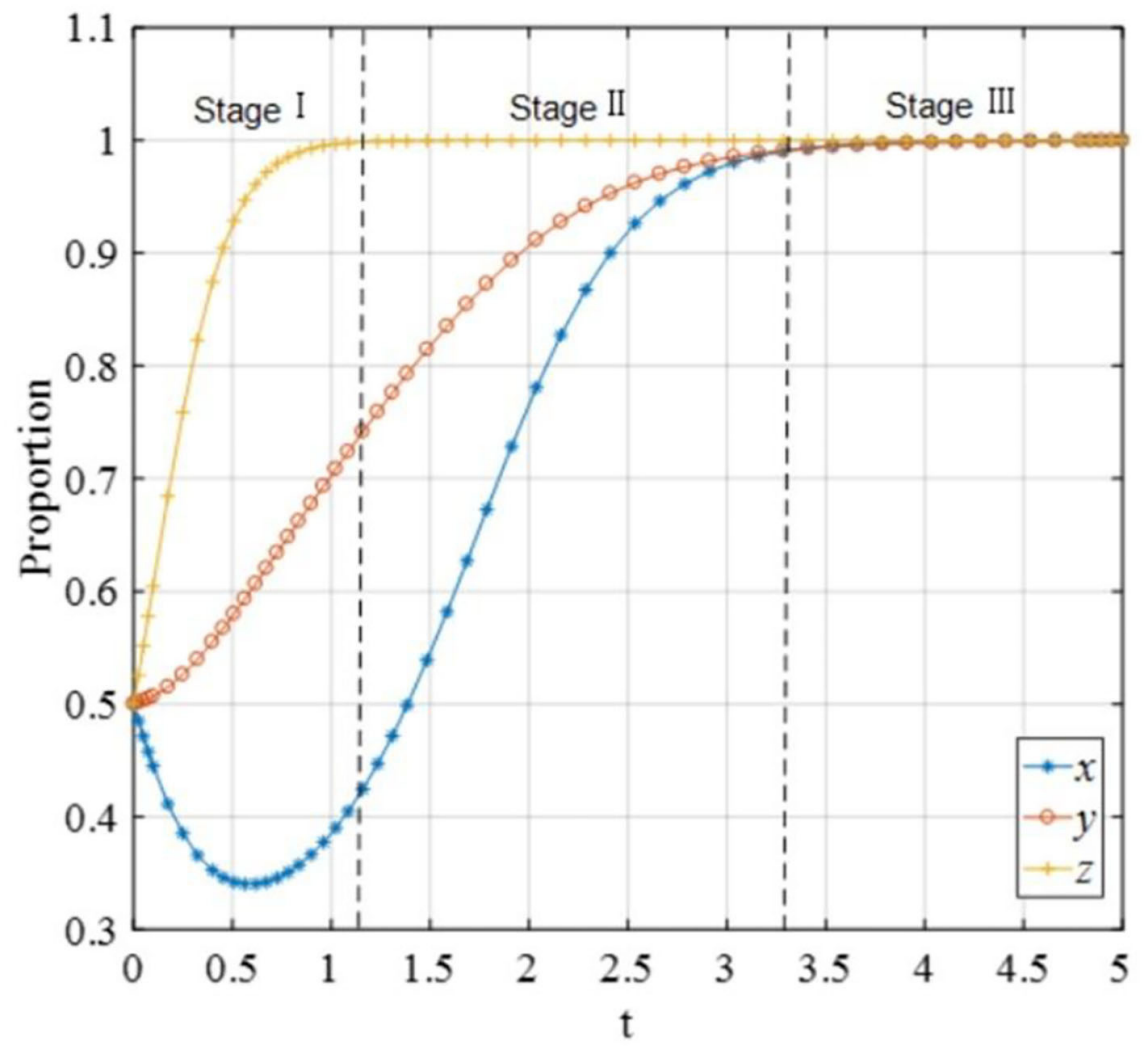
The evolutionary process with the co-governance scheme.

The final evolutionary stable strategies are ultimately unchanged when the initial state changes and other parameters are fixed. Regardless of the initial ratio of strategies adopted by P2P lending platforms, investors, and financial regulators, the final ESS would be *E*_8_(1, 1, 1). This result indicates that the initial states of the three players are not related to the initial proportions, but their initial proportions would affect the evolutionary speed. The higher the proportions of P2P lending platforms adopting the “positive disposal” strategy, the investors adopting the “participating in co-governance” strategy, and the financial regulators adopting “co-governance regulation,” the swifter the game would converge to *E*_8_(1, 1, 1). This reveals the necessity to build up the awareness of investors to participate in this co-governance pattern. In particular, the financial regulators should pay more attention to the P2P lending platforms with the low initial willingness of adopting “positive disposal” and enhance their supervision on those platforms. Above all, the financial regulators are expected to implement the co-governance regulation strategy to protect the rights and interests of the investors, in order to accelerate the P2P lending transformation and enhance the sustainable development of the FinTech industry. Specifically, we divide the overall evolutionary process into three stages according to the convergence states of the three players, as shown in Figure 16.

#### Stage I, the initial stage

At this stage, the proportion of financial regulators choosing “co-governance” presents an upward trend, while the proportion of investors choosing “co-governance” is on the rise and reaching a stable state. Since the initial stage of the establishment of a benign exit scheme of P2P lending platforms, financial regulators need to play the leading role of co-governance as much as possible to prevent the negative exit of the P2P lending platforms from damaging the rights of investors. However, due to the hysteresis of benign exit schemes and the legal of P2P lending platforms, the proportion of P2P lending platforms choosing “positive dispose” has gone through a process of first reducing and then increasing. At the same time, the enforcement and supervision of the benign exit of the P2P lending platforms are not mature enough, thus financial regulators should focus on improving the participation of investors in the benign exit process and establish regulatory norms to promote the benign exit process of the P2P lending platforms mainly by establishing regulatory norms.

#### Stage II, the transitional stage

The proportion that the P2P platform chooses to actively dispose of will rise to 1 as the proportion of the financial supervision department choosing co-governance supervision increases, and the proportion that the investor chooses to participate in co-governance is 1 and remains stable. At this stage, since the P2P lending platforms received penalties from financial regulators and social reputation loss from investors, they will have the consciousness to support the benign exit process actively. However, the enforcement and supervision of the benign exit process is a long-term and arduous task, a co-governance pattern driven by financial regulators has been gradually formed in the benign exit of P2P lending platforms aimed at resolving conflicts of interest between the investors and P2P lending platforms. Thus, regulatory norms and value reconstruction will affect the behavior of stakeholders to participate in the co-governance in the benign exit of P2P lending platforms. To some extent, it demonstrates that co-governance of the financial regulators is still a guarantee for keeping P2P lending platforms to choose the strategy of “positive disposal.”

#### Stage III, the mature stage

We can find the proportion of P2P lending platforms choosing “positive dispose,” the probability of investors choosing “co-governance” and the probability of financial regulators choosing “co-governance” all remained stable at 1. At this stage, P2P lending platforms, investors, and financial regulators are ultimately stable and will not change their strategies due to the stable payoff, and the financial regulators can also avoid generating more sunk costs. The financial regulators and investors will collaborate to comply with the benign exit principles of P2P lending platforms, thus co-governance will become a by-product of the financial regulation. Various mechanisms for resolving conflicts of interest have been established at the level of the P2P lending industry to form an overall interest coordination mechanism for the benign exit of P2P lending platforms. Institutional norms and value reconstruction will affect the behavior of all stakeholders to participate in the co-governance of benign exit of P2P lending platforms. This has achieved the goal of benign exit of P2P lending platforms and formed a win–win situation for P2P lending platforms, investors, and financial regulators.

### Applicability of our model

The outline of the regulatory document “Opinions on the classified dispose and risk prevention of P2P lending platforms” regards the benign exit of P2P lending platforms as the top priority of regulation over P2P lending platforms and establishes a long-term mechanism for the FinTech loan industry. In fact, the co-governance of the benign exit of P2P lending platforms entails accelerating the exit and transformation of P2P lending platforms. This process will not only protect the legal rights and interests of investors but also drive one stream of P2P lending to be transferred into FinTech lending (Bussmann et al., 2020) in the emerging financial market. Thus, the necessary applicability and sustainability for the future development of the FinTech industry are provided.

However, at present, the benign exit process of P2P lending platforms is still faced with significant challenges. On the one hand, the benign exit lacks co-governance with the participation of investors, and it is excessively dependent on the regulation of financial regulators. On the other hand, the imperfection and hysteresis of the existing regulatory rules may lead to a lower level of efficiency in the benign exit process of P2P lending platforms, which would cause huge property losses to investors. In addition, in the process of benign exit, P2P lending platforms need to bear the high costs of asset disposals, while at the same time they are faced with difficulties in the operation process and often lack industry self-discipline. For investors, the lack of necessary funds to participate in the co-governance of benign exits of P2P lending platforms could lead to lower payoffs than expected. Therefore, all the stakeholders would have incentives to choose different strategies. For this reason, the strategies of the stakeholders are analyzed from the perspective of a tripartite evolutionary game, within which a truthful interpretation of the strategic choices is provided in the model results.

## Conclusion

This study established a tripartite evolutionary game model for the first time to discuss the co-governance pattern in the benign exit process of P2P lending platforms in China. The main conclusions are as follows. (1) There are eight equilibrium points in the tripartite evolutionary game model, but only four ESSs. From the perspective of theoretical analysis and numerical simulation, the equilibrium point *E*_8_(1, 1, 1) is a more appropriate choice for the co-governance scheme of benign exit of P2P lending platforms, i.e., the one where P2P lending platforms adopt the “positive disposal” strategy, investors adopt the “participating in co-governance” strategy and financial regulators adopt the “co-regulation” strategy, respectively. (2) The initial proportion of P2P lending platforms adopting the “positive disposal” strategy, investors adopting the “participating in co-governance” strategy, and financial regulators adopting the “co-regulation” strategy cannot change the ultimate ESS, but will affect the evolutionary speed, the higher the initial proportion is, the faster the evolutionary speed will be. (3) Certain factors play a vital role in the strategic choices of P2P lending platforms, investors, and financial regulators. Specifically, the “positive disposal” proportion of P2P lending platforms increases with the increase in a penalty given by financial regulators to P2P lending platforms and the reputation losses of P2P lending platforms. While the “positive disposal” proportion of P2P lending platforms increases with the decrease in total costs of P2P lending platforms. Moreover, the threshold effect exists in the rewards given by financial regulators to investors, the cost of investors when adopting the “participating in co-governance” strategy, and the investment volume of investors, which may change the evolutionary stable strategies of P2P lending platforms, investors, and financial regulators.

Based on the above results, related policy strategies are proposed. (1) The financial regulators should adopt the co-governance pattern in the benign exit process of P2P lending platforms, to guarantee that P2P lending platforms and investors are able to obtain optimal benefits. This would help facilitate the benign exit process of P2P lending platforms and provide deeper insight into the mechanism of transformation in the FinTech industry. (2) The financial regulators should leverage regulation technology (RegTech) to reduce the initial costs of building the co-governance pattern and encourage investors to raise their awareness of participating in the benign exit process. (3) At the beginning of the benign exit of the P2P lending platforms, priority should be given to the willingness of P2P lending platforms that choose the “benign exit” strategy and investors who choose the “participating in co-governance” strategy. By doing so, we can effectively prevent capital losses and increase the probability of the benign exit of P2P platforms. (4) When P2P lending platforms choose a “benign exit” strategy, but the willingness of investors to choose “participation in co-governance” is low, the regulators can effectively address this problem by imposing higher penalties on P2P lending platforms and improving the incentives for investors.

## Data availability statement

The raw data supporting the conclusions of this article will be made available by the authors, without undue reservation.

## Ethics statement

Ethical review and approval were not required for this study as the game models in this paper are based on hypotheses and numerical simulations, not real experiments. The “participants” in this paper are all simulated parameters, not patients/participants who actually participated.

## Author contributions

QW and XL: conceptualization, methodology, and writing—review and editing. XL: software. QW: writing—original draft preparation. CZ: supervision and project administration. All authors contributed to the article and approved the submitted version.

## Footnotes

^1^The Regulation and Rectification Office (RRO) is affiliated with China Banking and Insurance Regulatory Commission, which is primarily responsible for preventing and rectifying the risks of Internet finance and P2P lending industry.

^2^See http://www.nifd.cn/ResearchComment/Details/1229
